# Improving future agricultural sustainability by optimizing crop distributions in China

**DOI:** 10.1093/pnasnexus/pgae562

**Published:** 2025-01-07

**Authors:** Qi Guan, Jing Tang, Kyle Frankel Davis, Mengxiang Kong, Lian Feng, Kun Shi, Guy Schurgers

**Affiliations:** State Key Laboratory of Lake Science and Environment, Nanjing Institute of Geography and Limnology, Chinese Academy of Sciences, Nanjing 211135, China; School of Environmental Science and Engineering, Southern University of Science and Technology, Shenzhen 518055, China; Department of Geosciences and Natural Resource Management, University of Copenhagen, DK-1350 Copenhagen, Denmark; Center for Volatile Interactions, Department of Biology, University of Copenhagen, DK-2100 Copenhagen, Denmark; Department of Physical Geography and Ecosystem Science, Lund University, SE-223 62 Lund, Sweden; Department of Geography and Spatial Sciences, University of Delaware, Newark, DE-19716, USA; Department of Plant and Soil Sciences, University of Delaware, Newark, DE-19716, USA; Eastern Institute for Advanced Study, Eastern Institute of Technology, Ningbo 315200, China; School of Environmental Science and Engineering, Shanghai Jiao Tong University, Shanghai 200240, China; School of Environmental Science and Engineering, Southern University of Science and Technology, Shenzhen 518055, China; State Key Laboratory of Lake Science and Environment, Nanjing Institute of Geography and Limnology, Chinese Academy of Sciences, Nanjing 211135, China; Department of Geosciences and Natural Resource Management, University of Copenhagen, DK-1350 Copenhagen, Denmark

**Keywords:** crop switching, fertilizer management, climate change, agricultural sustainability, national scale

## Abstract

Improving agricultural sustainability is a global challenge, particularly for China's high-input and low-efficiency cropping systems with environmental tradeoffs. Although national strategies have been implemented to achieve Sustainable Development Goals in agriculture, the potential contributions of crop switching as a promising solution under varying future climate change are still under-explored. Here, we optimize cropping patterns spatially with the targets of enhancing agriculture production, reducing environmental burdens, and achieving sustainable fertilization across different climate scenarios. Compared with current cropping patterns, the optimal crop distributions under different climate scenarios consistently suggest allocating the planting areas of maize and rapeseed to the other crops (rice, wheat, soybean, peanut, and potato). Such crop switching can consequently increase crop production by 14.1%, with accompanying reductions in environmental impacts (8.2% for leached nitrogen and 24.0% for irrigation water use) across three representative Shared Socio-economic Pathways from 2020 to 2100. The sustainable fertilization rates vary from 148–173 kg N ha^−1^ in 2030 to 213–253 kg N ha^−1^ in 2070, significantly smaller than the current rate (305 kg N ha^−1^). These outcomes highlight large potential benefits of crop switching and fertilizer management for improving China's future agricultural sustainability.

**Significance Statement**

Under future food demand increases and rapid climate change, attaining greater agriculture production with reduced environmental impacts is one of the grand challenges in China's cropping systems. We assess the benefit potentials of crop switching and fertilizer management under varying future climate scenarios, and thereby identify that optimizing cropping spatial patterns under future climate can generate markedly increased crop production with reduced environmental costs. Furthermore, the current fertilization rate is at nearly twice sustainable boundaries in 2030, implying that reducing and redistributing nitrogen usage can better ensure a sustainable future for China's agriculture ecosystems. Our study provides compelling strategies for promoting future food security and achieving Sustainable Development Goals in China.

## Introduction

Over the past half-century, rapid population growth and unprecedented economic development have driven remarkably rising food demand in China (1), leading to tremendous pressure to enhance national crop production (CP) with only 8% of global arable land and 5% of global freshwater resources (2, 3). Since the 1980s, several conventional and efficient measures have been extensively implemented to stimulate CP at the national scale, such as cropland expansion (4), intensified nutrient usage and irrigation (5). For example, China's fertilizer applications increased from 13.4 Mt N in 1985 to 29.1 Mt N in 2010 (5), along with advanced agricultural technologies and irrigated cropland expansion (4), almost achieving doubled CP (from 382 Mt in 1987 to 651 Mt in 2010) (3) and nearly reaching self-sufficiency for three staple crops (rice, wheat, and maize) at the national level (6). However, the current cropping patterns in China are overall smallholder-regulated systems with considerable variability under the conventional practice-based management by millions of farmers (7). Rice, maize, and wheat dominate high productive agriculture zones (the North and Northeast China Plain, as well as the Yangtze Plain) across the entire country (6), forcing soybean to grow in the limited area and thereby rely heavily on importation (8). Widespread cultivation of maize in arid regions, such as the northern and northwestern China, consumes substantial irrigation water withdrawn from groundwater (9), and concomitantly escalates groundwater depletion and land subsidence (10). Excessive nitrogen applications (average to 305 kg N ha^−1^ yr^−1^, refs (11)) become agriculture ecosystems as high-input, low-efficiency farming systems, lowering nation-averaged nitrogen use efficiency (NUE, the ratio between total nitrogen input and harvested nitrogen) to 0.25 in 2010 that is much lower than the values of 0.42 worldwide and 0.65 in North America (12). Moreover, the current cropping patterns are vulnerable to climate change, and always suffer from the penalty of agriculture production (13, 14). In recognition of these environmental tradeoffs, achieving agricultural benefit–cost balances with high production and low environmental impacts is urgent and critical for ecologically and societally sustainable development.

China's food demand is projected to increase markedly in the near future (2010–2050), with the necessity of further increasing agricultural productivity and promoting future food security (15). However, widespread climate change, such as temperature increase, intensified drought and flooding, as well as heatwave events, will challenge efforts to maintain or enhance future agriculture production (16–19). Rising temperature and carbon dioxide (CO_2_) concentrations in the atmosphere could enhance photosynthetic rates and thereby stimulate crop yield in temperate and cold zones (16, 19). For the southern parts of China, the more frequent heatwaves might increase the risk of crop death and yield failures by impairing photosynthesis systems and intensifying respiration rates (20). The projected changes in precipitation patterns (21), especially in the anthesis and grain-filling periods that are critical for crop development, would alter carbon assimilation and allocation, and potentially introduce yield failure and loss in agriculture ecosystems (17). Moreover, the intensified extreme rainfall and flood events can temporarily promote water availability, which might force crops to grow under anoxic conditions with the ultimate reduction in agriculture production (22). In this regard, the impacts of the changing climate on agriculture ecosystems are necessary to consider when sustainably enhancing agricultural productivity and promoting future food security.

Under future climatic conditions, agricultural benefits in response to historical practices would potentially diminish as productivity enhancement and the severity of environmental impacts close to their attainable boundaries (23). Instead, crop switching that redistributes cropping patterns spatially might be a promising option to improve agricultural sustainability with the overall benefits of greater productivity and lower environmental impacts. Previous regional and global studies have proven that optimizing crop distributions spatially can yield clear benefits on agriculture productivity, resource (nitrogen and water) use efficiency, climate resilience and biodiversity, especially in India and China that are the vast smallholder-farming communities (24–27). Under this condition, the Chinese government seeks to improve agriculture sustainability through mobilizing smallholder farmers to optimize cropping patterns with scientific guidelines and incentive policies, such as high-standard farmland and water-saving projects (2, 11). A recent study that focuses on crop switching of 13 crops at the national scale could provide a feasible scheme for sustainable development in agriculture because crop switching can realize co-benefits for environmental sustainability and farmer incomes in China under increasing levels of government coordination (25). However, such an assessment that focuses on historical climatic conditions and management practices cannot reflect the effects of varying climate change and incentive policies to crop-switching contributions to improving future agricultural sustainability. Although there exist several pioneering researches showing that crops can become more adaptable and productive at regional to national scales after reallocation of crop planting area and optimization of crop planting date in response to future climate change (8, 13, 14), they tend to focus on few representative crops, such as maize and soybean, lacking a comprehensive picture across different crops and their overall impacts on the balance between agriculture production and environmental costs.

Here, we optimize spatial distributions of seven main staple crops represented by 11 crop functional types (CFTs) that grow in 81.5% of total cropland area and harvest 97.5% of total CP in China (28), and assess their improvements on agricultural sustainability, simultaneously promoting national food security, and reducing environmental impacts under varying climate change scenarios in the 21^st^ century (Fig. S1). Using gridded agro-ecological suitability data simulated by the LPJ-GUESS model, we conduct multiobjective optimizations with three sustainability objectives (i.e. maximum of CP, minimum of leached nitrogen (LN), and minimum of irrigation water use [IWU]) and three key constraints (i.e. promoted self-sufficiency in crop demand, unaltered cropland area within each county, and minimized effects on supply chain), and then compare with the baseline scenarios to quantify the contributions of crop switching to future sustainable agriculture across the entire China under varying climate change and fertilizer management. We assume that: (i) crop switching is exempt from the present and future food trade in the optimization of crop distributions; (ii) the current cropland extent is unaltered under baseline and future climate change scenarios; and (iii) sufficient irrigation water resources withdrawn from groundwater can ensure crop growth without water stress in the simulations of agro-ecological suitability maps. With the optimal crop distributions, we also simulate the response functions of agriculture outputs (i.e. CP and LN) to fertilization variations, and discuss how to achieve sustainable fertilizer management in the near- (2030), mid- (2050), and long-term (2070) futures. Such assessments not only underscore the pivotal roles of crop switching and fertilizer management in improving future agricultural sustainability, but also serve as supporting information for policymakers to formulate agricultural guidelines and management practices.

## Results

### Simulations of future agriculture outputs

**Fig. 1.**
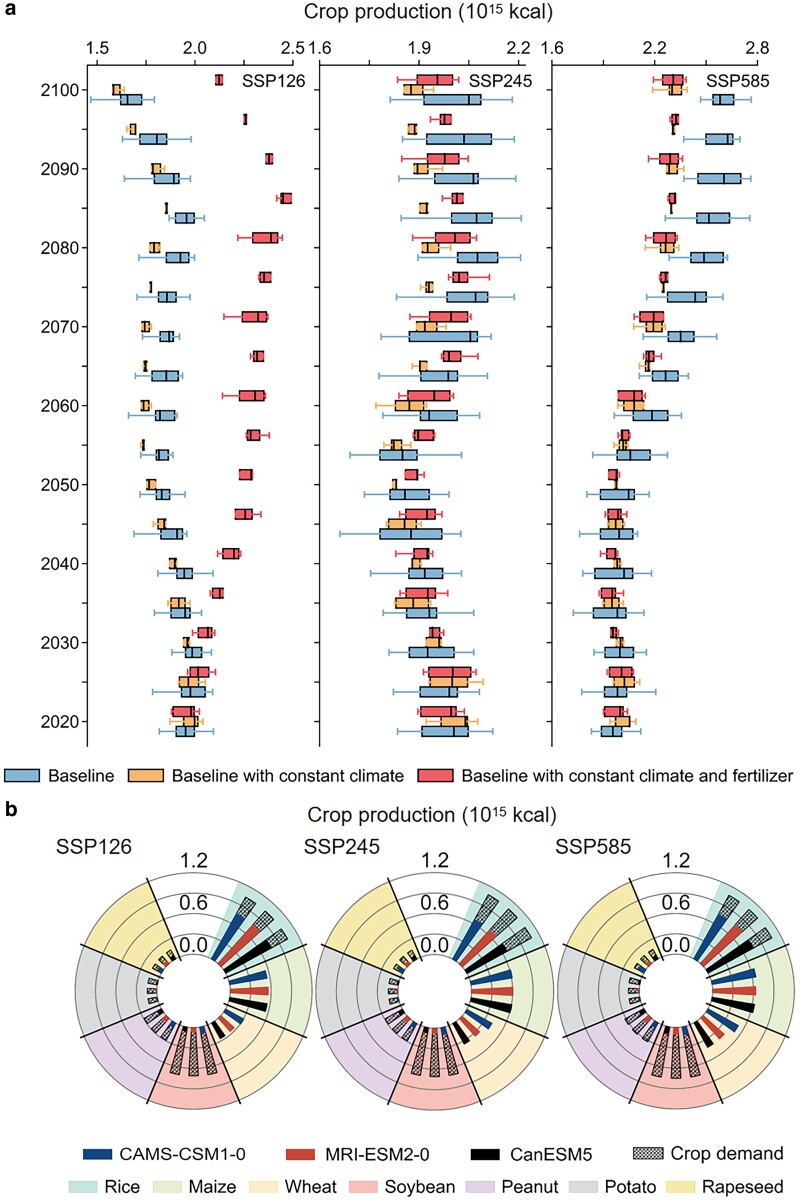
Future CP driven by climate change and fertilizer management. a) Comparison of CP changes across three different scenarios that include the “Baseline” (historical crop distribution, but future climate and fertilizer application), “Baseline with constant climate”, and “Baseline with constant climate and fertilizer” scenarios. The constant climate is obtained by recycling climate data in 2005–2014 over the future period (i.e. 2015–2100) to drive the LPJ-GUESS simulations, and the constant fertilization rates were obtained by recycling the fertilization rates from 2005 to 2014. b) Crop-specific supply–demand gaps under different SSPs and GCMs. The colored bars show CP variability between three examined SSPs, while the hatched bars represent crop demand. Here we regarded the sum of rice, wheat and maize production as cereals (hatched bars in rice panels).

We used the dynamic ecosystem model LPJ-GUESS (29, 30) to simulate historical and future agriculture outputs (CP, LN, and IWU) in response to climate change and projected fertilization (Figs. S2 and S3) in China (see Methods). Evaluation performance showed high accuracy levels on crop yield, the length of growing seasons and two vegetation-related indicators for the historical period (1979–2014) (Fig. S4). The model simulated steady CP from 2015 to 2100 under SSP126 and SSP245, and consistently increasing CP (+0.70 × 10^15^ kcal, or +36.3% from 2015 to 2100) under SSP585 when maintaining historical crop distributions with varying climate change and fertilizer applications as the baseline scenario (Fig. 1a). Over 86% of total CP was attributed to the three main crops (rice, wheat, maize). Consequently, these three main crops achieved self-sufficiency at the national level, while the large supply–demand gaps were observed for the other four crops studied here, particular for soybean with CP being only 13% of crop demand (Fig. 1b). Climate warming and fertilization can certainly contribute to enhanced CP; their associated increasing rates were 0.02 to 0.05 × 10^15^ kcal °C^−1^, and 0.31 to 22.88 × 10^12^ kcal Mt^−1^ across three different socio-economic pathways (SSPs), respectively (Fig. 1a). The shapely declining fertilization restrained agriculture productivity, resulting in continuous degradation in CP under SSP126, while climate warming exerted a strong impact on CP under SSP245 (Figs. 1a and S3). The declining fertilization was also accompanied by continuous LN decrease by 39 to 86% from 2015 to 2100, respectively (Fig. S3), which was accompanied by increased NUE (+0.11 to +0.29) across three different SSPs. Nevertheless, the rising crop demand and supply–demand gaps still require achieving increased agriculture productivity without extra environmental costs. In addition, the IWU demand was simulated to increase for low-level warming scenarios (Fig. S5).

### Overall benefits of optimization in crop distributions

**Fig. 2.**
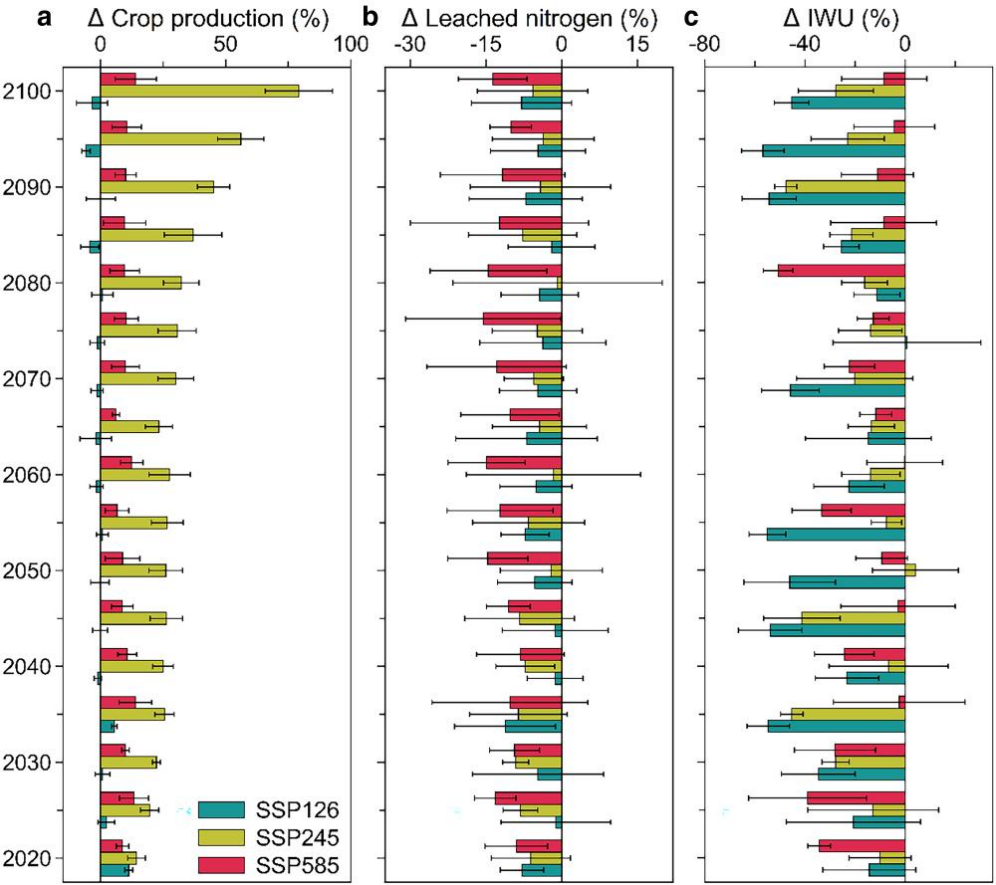
Overall optimization benefits for agriculture productivity and environmental impacts. Optimization benefits were calculated as the difference in three dimensions, including CP (a), LN (b), and IWU (c) between the optimal and baseline crop distributions, where the baseline scenario was assumed as historical crop distributions under each SSP. The error bars show 1 SD among three GCMs.

Compared with the baseline levels from 2020 to 2100, optimizing crop distributions can generate co-benefits for enhanced agriculture productivity (CP increase by 14.1% ± 16.2%) and reduced environmental impacts (LN reduction by 8.2% ± 3.7% and irrigation water savings by 24.0% ± 17.1%) (Figs. 2 and S6). The production increases in wheat, peanut, and rapeseed contributed dominantly (88.5%) to total optimization-induced benefits on CP, while environmental impact reductions were primarily attributed to maize with LN decreased from 1.70 ± 0.59 Mt N to 0.51 ± 0.58 Mt N, and IWU decreased from 19.78 ± 7.47 km^3^ to 14.44 ± 5.65 km^3^ across all three SSPs (Fig. S7). Considerable variations characterized these overall benefits between the different SSPs, mostly associated with different fertilization and climate change rates. The largest gain in CP was examined under SSP245, with the net increase of 32.2% ± 15.1% from 2020 to 2100, indicating the evolving roles of crop switching in enhancing CP under moderate warming climate.

**Fig. 3.**
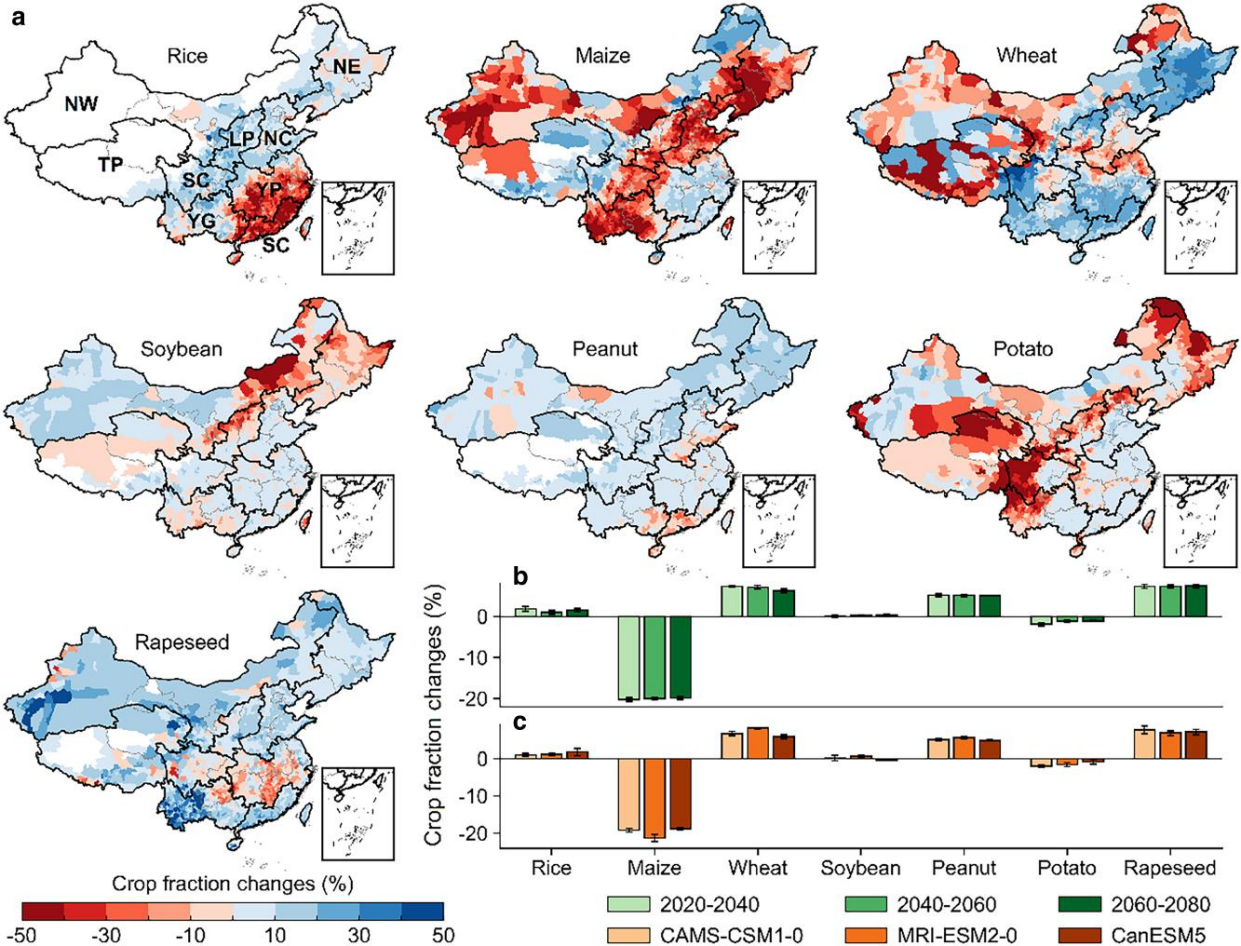
Optimized changes in crop distributions on national scales. a) Spatial variations in county-based crop fraction (defined as the fraction of crop-sown area over total cropland area) changes (rice, maize, wheat, soybean, peanut, potato, and rapeseed), represented by average crop distributions of three SSP245 GCMs throughout the entire future period. Nice regions of China are also mapped here: NE, Northeast China Plain; LP, Loess Plateau; NC, North China Plain; YP, Yangtze Plain; SC, Southern China; NW, Northwestern China; TP, Tibet Plateau; SC, Sichuan Province; YG, Yun-Gui Plateau. b) Overall crop-specific fraction changes for three sub-periods (2020–2040, 2040–2060, and 2060–2080). The error bars present 1 SD in each sub-period. c) GCM-related variability in crop fraction changes for the example period of 2040 to 2060. Similarly, the error bars also represent one standard deviation between different SSPs.

**Fig. 4.**
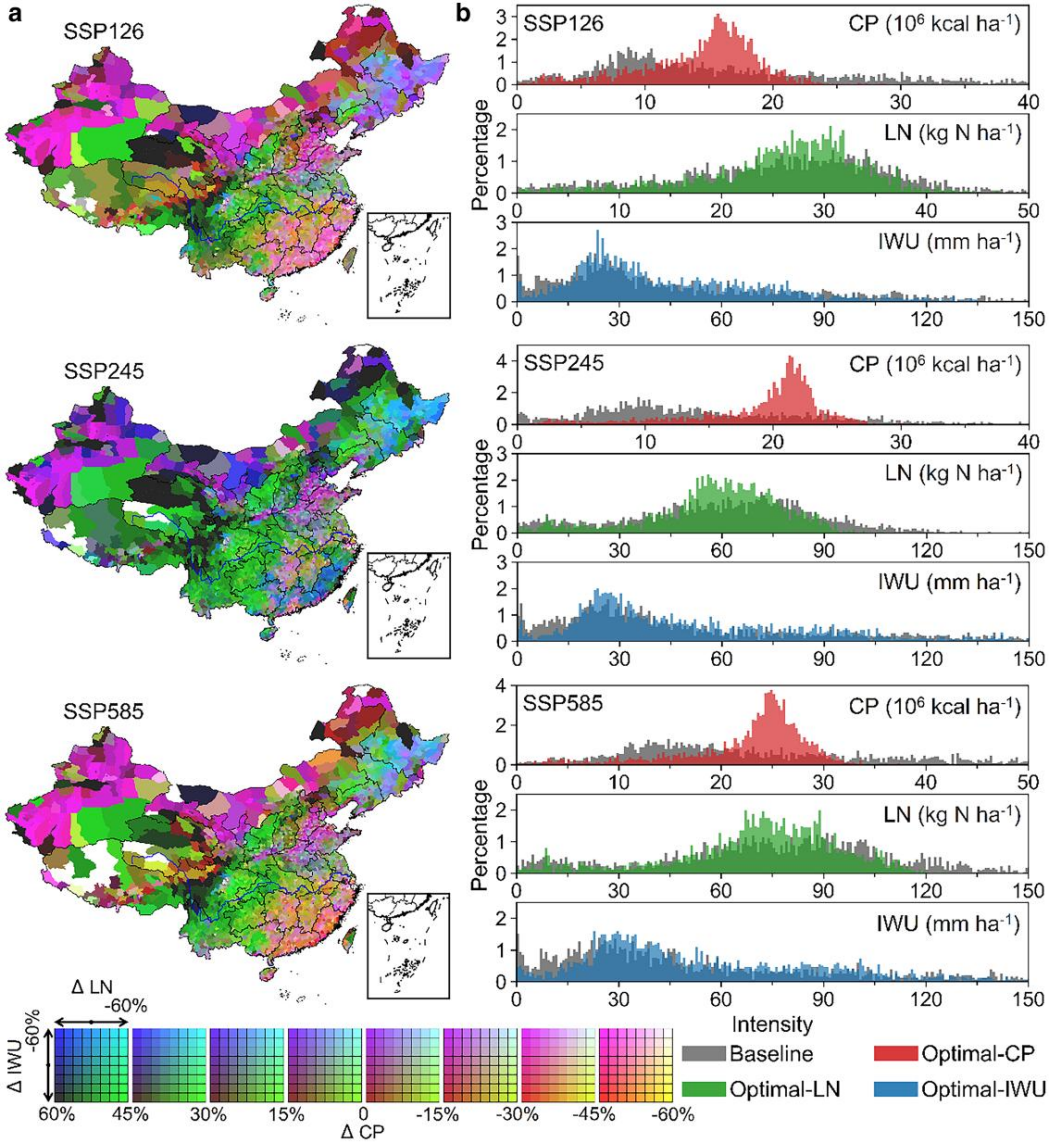
Spatial variability of optimization benefits. a) Spatial patterns of relative changes in CP, LN, and IWU relative to the baseline values for the future period. Each panel represents relative changes in LN and IWU at the given magnitude in CP changes. b) Histogram of baseline and optimal CP, LN, and IWU intensity that was calculated as the ratio of three indicators against the sown area (unit: 10^6^ kcal ha^−1^ for CP, kg N ha^−1^ for LN, and mm ha^−1^ for IWU).

All crop switching-induced benefits were achieved by reallocating crop-sown area (i.e. spatial distributions and areal fractions) across the entire China (Figs. 3 and S8). We observed that the optimized crop switching patterns were similar across the different GCMs and periods (Fig. 4b and c), where the optimal crop distributions under SSP245 generally allocated the sown area of maize (−19.8%) and potato (−1.2%) to rice (+1.0%), wheat (+7.2%), soybean (+0.3%), peanut (+5.2%), and rapeseed (+7.3%) for the period of 2040–2060 (Fig. 3b). Specifically, the overall sown area changes in maize dominantly contributed to national cropland reallocation, with the declining sown area in three main agricultural regions (southwestern China, the North, and Northeast China Plain), which actually follows the recently launched National Planning on Crop Structure Adjustment that aims to sustainably meet the domestic nutrition demand through reducing the maize-sown area (2). Rice-sown hotspots shifted northwards from the Yangtze Plain to the North and Northeast China Plain (Fig. 3a). Furthermore, the northern Northeast China Plain experienced the expansion of sown area of wheat and peanut; the Yangtze Plain exhibited the decline in rapeseed planting area replaced by other crops (see Figs. 3a and S8).

### Spatial variations in three optimization dimensions

Considerable regional variations characterized benefit patterns across all three optimization dimensions, suggesting that crop switching can achieve sustainable co-benefits at the national level accompanied by varying tradeoffs between different dimensions and regions. Ideally, the sustainable co-benefits, defined as enhanced CP and reduced environmental impacts, however, only occurred in 4.0 to 9.2% of national counties and exclusively emerged in the Northeast China Plain across three examined SSPs (Fig. 4a). The remaining regions were generally characterized with inevitable tradeoffs. For instance, enhanced CP and decreased LN at the higher IWU cost were widely observed in high productive and populated regions (31), such as the southwestern and central China, as well as the western parts of North China Plain, promising to meet the rising food demand in these regions (15). Conversely, irrigation water savings with the degradation of CP and the increase of LN primarily focused on the southern and northwestern China, as well as the most parts of North China Plain; these tradeoffs would still be valuable to mitigate maize-relevant irrigation water shortage and groundwater depletion in the North China Plain (32), though at higher risks in freshwater nutrient security. Lastly, although crop switching led to overall compromises for CP over the Yangtze Plain, the reduction in LN could lessen widespread eutrophication in freshwater ecosystems (33).

Tradeoffs between different optimization dimensions caused significant changes and variations in the distributions of CP and environmental cost intensity across three different SSPs (Fig. 4b). The largest magnitude in distribution changes was found for SSP245; associated magnitudes were +4.1 × 10^6^ kcal ha^−1^ for CP intensity, −3.4 kg N ha^−1^ for LN intensity, and −10.8 mm ha^−1^ for IWU intensity, respectively (Fig. 3b, SSP245). Furthermore, the proportion of high-production counties (CP intensity > 20 × 10^6^ kcal ha^−1^) increased by 39.4%, relative to the baseline level (20.0%), which was accompanied by the net proportion decrease of −13.2% and −7.7% in high LN- (> 75 kg N ha^−1^) and IWU-intensity (>90 mm ha^−1^) counties. Such distribution changes suggested that sustaining regional tradeoffs for CP and environmental costs can certainly achieve more sustainable agriculture systems nationally.

### Agricultural response to fertilizer management

**Fig. 5.**
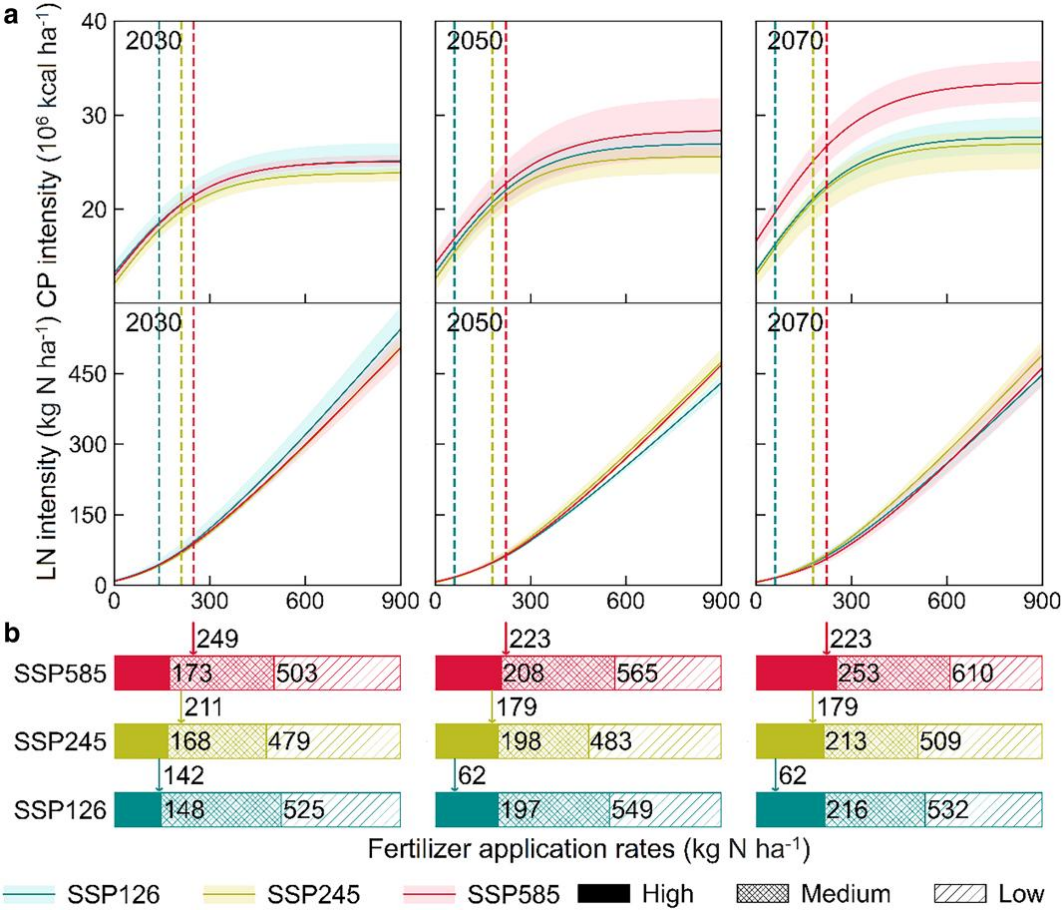
Agriculture response to future fertilizer management. a) Response curves of CP and LN intensity to nitrogen fertilizer application rates, estimated from optimal crop distributions in the near- (2030), mid- (2050), and long-term (2070) futures (see Methods). Colored shadows represent the response ranges of three different GCMs, and colored dash lines indicate CP and LN intensity to the baseline fertilization rates used in this study. b) Estimated national fertilization priority levels, defined with response rates of CP intensity (low, <5 × 10^3^ kcal kg N^−1^; medium, 5–30 × 10^3^ kcal kg N^−1^; high, >30 × 10^3^ kcal kg N^−1^). The baseline fertilization rates (colored arrows) and threshold rates of each fertilization priority were annotated here.

Sustainable fertilizer management is an important aspect of agricultural sustainability, especially in China's high-fertilization cropping systems. Realizing sustainable fertilization goals, aiming to maintain high agriculture productivity without much nutrient exports into soils and freshwater, requires detailed crop distributions and associated agricultural response (34). With the county-based optimal crop distributions, we simulated agriculture outputs (CP and LN intensity) to varying fertilization rates and then fitted their response curves through logistic and polynomial functions in the near- (2030), mid- (2050), and long-term (2070) futures (see Methods). Consequently, climate change promoted attainable CP intensity from 24.7 × 10^6^ kcal ha^−1^ in 2030 to 29.4 × 10^6^ kcal ha^−1^ in 2070 (Fig. 5a). We partitioned the response curves into three different stages of fertilization priorities based on the response rates for CP intensity, low (<5 × 10^6^ kcal kg N^−1^), medium (5–30 × 10^6^ kcal kg N^−1^), and high (>30 × 10^6^ kcal kg N^−1^) productivity, and then regarded high fertilization priority as sustainable fertilization ranges due to the corresponding high agricultural response and low-ratio nitrogen leaching from fields. Under these conditions, sustainable fertilization co-boundaries were recommended to be 148–173 kg N ha^−1^ in 2030, 197–208 kg N ha^−1^ in 2050 and 213–253 kg N ha^−1^ in 2070 as national average levels of all three SSPs (Fig. 5b), where nationally total sustainable fertilization (23.1 ± 1.8 Mt N) in 2050 is in accordance with the previous estimate (27 Mt N) that follows the FAO's targets of agriculture productivity and sustainability (12). However, our recommended fertilization boundaries in 2030 reduced fertilization rates with 46% relative to 2010, being almost equal to average rates (161 kg N ha^−1^) of the United States in 2011 (35), suggesting that much efforts to gradually decrease fertilizer applications are required to realize sustainability goals in future agriculture ecosystems.

Our baseline fertilization rates were mostly within the sustainable ranges, except for those slightly larger rates under SSP245 and SSP585 in 2030, SSP585 in 2050 (Fig. 5b). However, the current fertilization rates under SSP126 only accounted for 31.5%, and 28.7% of sustainable co-boundaries in the mid- (2050) and long-term (2070) futures, respectively, indicating the largely potential space for fertilization-induced enhancement in CP without clear environmental tradeoffs. Further fertilization up to sustainable boundaries can thereby gain CP intensity by as much as 4.9 × 10^6^ kcal ha^−1^ in 2050, and 6.0 × 10^6^ kcal ha^−1^ in 2070 under SSP126, though at the cost of LN intensity increased by 38.3 kg N ha^−1^, and 45.3 kg N ha^−1^, respectively. These increased CPs can feed 192.2 and 224.1 million more people. We also observed that further fertilization hotspots primarily focused on the northwestern China, the western Northeast China Plain, and the Yunnan Province (Fig. S9a), all of which easily suffered from regional limited fertilizer accessibility (36). In contrast, many historically highly productive regions were almost fertilization-saturated (>70% of applied fertilizer lost as LN in Fig. S9b), including the eastern Northeast China Plain, the northern North China Plain, and the southern China, further revealing the importance of regulating regional fertilizer accessibility to improving agriculture sustainability.

## Discussion

### Enhancing agriculture adaptation to climate change

We have revealed that optimizing crop distributions spatially can improve agriculture sustainability in response to future climate change and fertilizer management. Overall, three main staple crops (rice, maize and, wheat) contribute mostly to national crop switching and concomitant co-benefits under the future warmer world. The faster warming and summer precipitation increases in the northern China will become more agro-climatically suited for northward expansion of rice-sown area (6), and thus yield more with the less irrigation water demand (Fig. S7). We recommend to reduce the planting area of maize in semi- and arid regions, but increase in the wet that holds abundant freshwater resources (37). Such switching in maize can largely mitigate environmental burden without massive loss in crop yield. Compared with rice and maize, wheat is a more drought-tolerant crop (38), which may explain its vast contributions to irrigation water savings, especially in the Northeast China Plain that is projected to be drier under future climate changes (6). Simultaneously, the warmer climate can expand the growing seasons and thereby enhance the wheat productivity. The sown area of soybean primarily increases in the northern and northwestern China, as well as the Yangtze Plain because much more precipitation could relieve the limitation on soybean production (14). Moreover, potato is more susceptible to water stress than many others (39), suggesting that switching towards the wetter regions, such as the eastern and southern China, would potentially promote CP. Peanut and rapeseed expand to the entire country because the higher temperature makes the more arable area agro-climatically suited for these two crops, resulting in the benefits on CP despite the slight environmental costs. All crop switching can certainly enhance agricultural adaptation to climate change, and make future agriculture ecosystem more sustainable.

### Limitations and uncertainties

Several limitations and uncertainties are present in the model evaluation, prediction of future food demand, and optimization of crop distributions. Specifically, due to the limited availability of the observed data, we did not calibrate and evaluate the LN in the simulations. Instead, the overall model performance was evaluated based on vegetation dynamics and crop phenology, and concluded that the calibrated model can simulate accurate vegetation dynamics in agriculture systems. Moreover, nation-averaged NUE was 0.25 in 2010 (12), close to our simulated value (0.27). Nevertheless, we acknowledge that some uncertainties remain in the simulations of LN.

We predicted future food demand using the recalibrated empirical relationships between GDPs and per capita caloric demand, suggesting that the diet transition varied with GDPs under future climate scenarios. However, there are many other drivers to regulate human dietary, such as the culture, nutritional knowledge, and taste preference (40). Such dependences may generate uncertainties in predicting future food demand. The diet of feeding animals was assumed to be unaltered in the conversion of animal food demand to the associated crop caloric demand, possibly introducing uncertainties because the diet might modify with the advance of feeding technology (41). Furthermore, the optimal crop switching focused on a total of seven main staple crops, without considering vegetables, fruits, and sugarcane due to their small proportion (18.5%) of total cropland area (42), which impeded to understand the contributions of these crop switching to achieve sustainable agriculture at the national scale.

We assumed that crop growth is exempt from water deficit in the growing seasons, possibly leading to unrealistic increases in irrigation water demand in arid regions. Although the county-based weights for terrestrial water availability were used to avoid these issues, more efforts are deserved to demonstrate the impacts of water availability to crop switching under future climate conditions.

### Implications for sustainable agriculture

Crop switching is a grand challenge for agriculture ecosystems in China that are currently dominated by smallholder management, because it requires vast amounts of investments and efforts from governments, farmers, and markets (8). However, there is a recent project that mobilizes millions of farmers to pursues sustainable productivity in China's agro-ecological zones (totally 452 counties) by adopting science- and evidence-based agricultural practices, such as changing crop types, optimizing fertilization strategies, and adjusting planting density (43). This project consequently yields CP increases by 10.8–11.5%, nitrogen fertilizer savings of 14.7–18.1% and the associated reduction in greenhouse gas emissions of 13.7–22.23%, in massive support of governmental subsidies, incentive policies, and specialized knowledge from 2005 to 2015 (7). It inspires us that crop switching at the national scale can be possible to realize when the coordinated actions that combine institutional knowledge, participatory strategies and political commitments by the Chinese central government starts.

Our study addresses sustainable fertilization management in future agriculture ecosystems, and recommends sustainable fertilization rates for different climate scenarios. In China, the main agricultural regions, such as the Yangtze Plain and the North China Plain, are generally characterized by high-fertilization rates and productivity, making them vulnerable to fertilizer over-usage and local nutrient-related issues over the soil and freshwater systems (44). However, less-developed and remote regions easily suffer from fertilization shortages and thereby limit the promotion of agriculture productivity (Fig. S9). Therefore, regulating chemical fertilizer accessibility and consumption is urgent and effective to achieve nationwide sustainable fertilization in future agriculture ecosystems.

In this study, our optimized crop switching scheme could potentially serve as a solution of “high-standard farmland” at the provincial to national levels that can be collaboratively implemented to improve agriculture productivity without the extra cost of input use (i.e. fertilizer, irrigation water) (25). The approach frameworks utilized here can be also potentially replicable and applicable to better characterize sustainable agricultures in other regions, such as India and the Southeast Asia that retain large arable land and low NUE (12, 45). Furthermore, with the doubled global crop demand estimates for food, livestock feed, and biofuels in 2050 (46), agriculture expansion and intensification might be widely adopted to enhance CP (47). Our results regarding fertilization management could also potentially serve as valuable baseline information to promote sustainable agriculture intensification at regional to global scales.

## Methods

### Dynamic vegetation model, LPJ-GUESS

We used the latest version of LPJ-GUESS model (v4.1) to simulate agriculture ecosystems in response to climate changes, nitrogen fertilization, and crop distributions over the entire China in the historical (1979–2014) and future (2015–2100) periods. In the LPJ-GUESS model, we defined CFTs with a set of bioclimatic, physiological, and phenological parameters to describe their capabilities for survival, competition, productivity, and development (29, 48). Gridded fractions of cropland derived from global land cover products were used as external inputs to represent spatial distributions and temporal dynamics of cropland area. Agriculture ecosystems are fully human-regulated, thereby meaning that the external files regarding the relative fractions of CFTs are required to describe the planting area of individual crops.

In total, we simulated seven main staple crops (rice, maize, wheat, soybean, peanut, potato, and rapeseed) represented by 11 associated CFTs (Table S1), together accounting for 81.5% of total cropland area and 97.5% of total CP in China (28). Crop distributions were determined based on national crop survey dataset compiled by the China Meteorological Data Service Center and global crop-harvested area products (see detailed descriptions below). The growth of CFTs was simulated on a daily time step, with the start of a prevailing climate-dependent sowing date and the end of harvest date associated with specified heat sum requirements (48). LPJ-GUESS allocates daily net primary production of crops to different organs (leaves, stems, roots, and grains) dependent on crop types and crop development stage. A detailed description of the allocation schemes can be found in ref. (48).

Atmospheric deposition, biological fixation, external chemical fertilizer and manure applications provide nitrogen sources for crop growth, where the uptake nitrogen is calculated as the minimum of crop demand and available soil mineral nitrogen (Supplementary Note S1). Meanwhile, both organic and inorganic nitrogen can leach from fields, potentially resulting in substantial nitrogen discharge into freshwater ecosystems. The leached organic nitrogen is affected by the decay rates of soil microbial carbon and soil percolation, while the leached inorganic nitrogen shows dependence on availability of soil mineral nitrogen, soil percolation and water content in soil columns.

Agriculture irrigation provides favorable water conditions for crop growth. In the LPJ-GUESS model, sufficient water resources are assumed to be withdrawn from surface and groundwater to fully satisfy irrigation water demand in cropland, suggesting that crop growth is not limited by water deficit and stress. We calculated IWU as the difference between available soil water content and water demand during the growing seasons, as those used in previous studies (24, 49, 50). Crop water demand is defined as the nonwater-stressed evapotranspiration (ET) rate, and calculated as a function of the potential canopy conductance (*g_p_*) and total equilibrium ET (*E_q_*). Irrigation water demand is updated daily, and then accumulated over the whole growing seasons as total IWU for crops. It is noted that such irrigation schemes would underestimate irrigation water demand for rice because they are irrigated by the continuous inundation (51).

### LPJ-GUESS gridded input data

LPJ-GUESS model requires five different gridded input data, which are climate data, soil properties, land use data, crop distributions, and fertilizer and manure applications for historical and future periods.

#### Climate data

We used daily climate data (temperature, precipitation, and short-wave radiation) from the China Meteorological Forcing Dataset (CMFD), with a spatial resolution of 0.1° to drive the simulations of the historical period (1979–2014) (52). For the projection of future climate, we selected monthly climate outputs from three general circulation models (GCMs), and three Shared SSPs in the latest CMIP6 project (53) to represent the different levels (high, moderate, and low levels) of future temperature and precipitation changes (Fig. S2). We performed a bias correction for the projections of future climate, calculated as the mean difference between the monthly CMFD and historical climate outputs from 1985 to 2014 as the biases (54), which was then added to future climate outputs (3 GCMs × 3 SSPs).

#### Soil properties

We derived soil properties (the fractions of sand, clay and silt, organic carbon content, C:N, pH, and bulk density) of each 0.1° grid cell from the World Inventory of Soil Property Estimates (WISE30sec) (55). The relative area of each FAO soil unit was calculated within one 0.1° grid cell, which were then used to extract the soil properties of the dominant FAO soil unit. We assumed the soil properties to be unaltered during the historical and future periods.

#### Land use data

We extracted the fraction of cropland from the Climate Change Initiative Land Cover (CCI-LC version 2.0) products for the period of 1992–2014 (56). This dataset contains totally 37 land cover types, where all crop-related land cover types were selected to represent cropland area (Table S2). The relative fractions of each class within 0.1° grid cells were then determined as input related to historical land use in China. Similarly, we used the land use data projected by Global Change Analysis Models (GCAMs) to force the future simulations from 2015 to 2100. The dataset has a spatial resolution of 0.05° and totally 39 land cover types (57). We also clustered all crop-related land cover types into cropland and thus determined its average relative fraction within each 0.1° grid cell. We used linear interpolation to derive annual land use data due to the 5-year time step of original dataset.

#### Historical crop distributions

We used global crop-harvested area products from 2000 to 2015 with a 5-year step and national crop surveys to derive the gridded cover fractions of CFTs (58). Firstly, the cover fractions of each crop were calculated as the ratios of its harvested area against total area of each 0.1° grid cell. Secondly, we used the cover fractions of potato, rapeseed, and peanut to calculate their fractional coverage. The other four crops (rice, maize, wheat, and soybean) contained multiple CFTs (Table S1), meaning that we should separate the cover fractions of each CFT based on national crop surveys and an adaptive inverse distance weighting method (59) (see details in Supplementary Note S2). Finally, we used linear interpolation to determine annual cover fractions of CFTs from 2000 to 2014, and assumed the cover fractions from 1979 to 1999 as constant values in 2000.

#### Fertilizer and manure application data

We extracted nitrogen fertilizer and manure application data from global nitrogen fertilizer and manure products for the historical period (60, 61). Considering the difference in spatial scales (0.5° fertilizer products and 0.05° manure data), we used the average manure within 0.1° grid cells and fertilizer application of the nearest grid cell to represent fertilizer and manure application rates, respectively. For the future period, the predicted fertilizer and manure applications decrease by 45, 20 and 6% from 2010 to 2030, and by 59, 14 and 9% from 2030 to 2050 under SSP126, SSP245, and SSP585, respectively (see Supplementary Note S3). We determined annual change rates using the linear interpolation, and then applied these estimates as spatially homogeneous across the cropland area in China. The fertilizer and manure application rates from 2050 to 2100 were assumed constant at the levels for 2050.

### LPJ-GUESS model calibration and evaluation

We performed the LPJ-GUESS model calibration and evaluation in the historical period (1979–2014). Observed crop yield data were collected from the provincial statistical yearbooks from 1979 to 2014, and then separated into the calibration (1979–1999) and evaluation (2000–2014) datasets. In the calibration process, we tested the different three-parameter combinations: default length of growing seasons, specific leaf area, and sowing temperature limits because these three parameters are critical to determine crop development stages and harvest decisions. We set the ranges of three parameters as 80–260 days, 30–70 m^2^ kg C^−1^ and 10–15 ℃, respectively. Crop yield for each CFT was simulated with each parameter combination (setting totally 37, 9, and 6 different levels for these parameters), and then compared with observed values. Finally, the parameter combination with the best performance (i.e. the lowest mean relative error (MRE) and the root mean square error) was selected to represent the CFTs in the LPJ-GUESS simulations.

Subsequently, the modeled results were evaluated based on the following key variables: crop yield, sowing and harvest dates, gross primary production (GPP), and ET. We derived point-based sowing and harvest dates from national crop surveys (58), and then compared with the simulated dates. Global Solar-induced Chlorophyll Fluorescence (GOSIF) GPP products from 2001 to 2014 (62) and Global Land Evaporation Amsterdam Model (GLEAM) ET products from 1980 to 2014 (63) were collected to evaluate the simulation of GPP and ET. Notably, we performed GPP and ET evaluation on grid cells with >80% cropland to minimize the mixed signals for other land use types. Our simulation overall agreed well with the evaluated data for all five variables (Fig. S4). We observed high accuracy of simulated crop yield, sowing and harvest dates across different crops, with the relative errors of < 15% (Fig. S4c). The simulated GPP and ET resulted in similar spatial distributions and low relative errors (20 and 14%) with the evaluated values.

### Baseline crop distributions for future simulations

We assumed historical crop distributions as the baseline scenario in the future simulations (3 SSPs × 3 GCMs). CP, LN and IWU were simulated to assess future agriculture productivity and environment costs under stable crop distributions in China.

### CP induced by climate change and fertilizer management

To separately quantify climate change and fertilizer management-induced CP, we designed two controlled scenarios: (i) “Baseline with constant climate” recycled climate data in 2005–2014 over the future period (i.e. 2015–2100); and (ii) “Baseline with constant climate and fertilizer” recycled climate data and fertilization rates in 2005–2014 over the future period, which were then compared with the baseline scenario to estimate the effects of climate change and fertilizer management to CP. Subsequently, the difference between the baseline scenario and two controlled scenarios was calculated as the climate change and fertilizer management-induced variability in CP under three different SSPs.

### Prediction of future crop demand

China's future crop demand was predicted using an empirical relationship between per capita calorie demand and real gross domestic product (GDP) that has been proven to perform satisfactory in China in previous studies regarding nationwide human-diet changes (40, 46). The calorie demand includes three different crop categories: (i) crop caloric demand (cereals, nuts, roots, and rapeseed), (ii) animal caloric demand (dairy and eggs, ruminant meat, poultry, pork, and seafood), and (iii) empty caloric demand defined as animal fats, oils sugars and alcohols (40). We collected GDP and the calorie demand, including crop caloric demand (cereals, nuts, roots, and rapeseed), animal caloric demand (dairy and eggs, ruminant meat, poultry, pork, and seafood) and empty caloric demand in the historical period of 1961–2009 from Food and Agriculture Organization (FAO) of the United Nations to recalibrate the empirical relationship (Fig. S10). As a result, these recalibrated relationships worked well, with the MRE of <25% (except for ruminant meat, 37.7%) and *R*^2^ of >0.9 (except for nuts, 0.6, and roots, 0.74). We then forecasted future per capita calorie demand based on the previously projected SSP-specific GDPs in the 21^st^ century (64). Our predicted per capita calorie demand agrees with the estimates through the numerical method (65) (Fig. S11). In addition, we converted animal caloric demand to associated crop caloric demand using feed-meat conversion rates (Table S3), making it accessible for determining the China's crop demand in the future period. Finally, we determined national crop demand as per capita crop caloric demand multiplied by the projections of total population (31). Our prediction of future crop demand was conducted with a 5-year time step that follows the availability of future GDP data.

### Mapping agro-ecological suitability

We defined the agro-ecological suitability of crops in this study as CP, LN, and IWU to document spatial difference in agriculture productivity and environment costs in the future period (2015–2100). It describes the attainable crop yields and associated impacts on soil and freshwater environment in response to biophysical conditions (such as climate, soil, and nutrient). We separately simulated the agro-ecological suitability maps for each CFT under the different climate scenarios (11 CFTs × 3 SSPs × 3 GCMs). Specifically, one given CFT was assumed to grow in all cropland area, meaning that its cover fraction was reset as one within each grid cell under each climate scenario. Such crop distributions were then used to drive the well-calibrated model to simulate the gridded CP, LN, and IWU from 2015 to 2100. Ultimately, these gridded agro-ecological suitabilities were integrated into county-based average values as the basic information for optimization of spatial crop distributions.

### Optimization of spatial crop distributions

We optimized county-based crop distributions based on an evolutionary many-objective optimization algorithm (non-dominated sorting genetic algorithm III, NSGA-III) and agro-ecological suitability data under all future climate scenarios. In this study, we focused on three conflicting objectives: (i) maximum of CP, (ii) minimum of LN, (iii) minimum of IWU over the entire China, and three constraints: (i) improving national self-sufficiency of CP, (ii) remaining unaltered total cropland area in each county, and (iii) minimizing CP loss in each crop against the baseline levels, ensuring the balance between CP and environment impacts for agriculture ecosystems. The optimization scheme can be expressed as follows:


$$\{\begin{array}{l} \begin{array}{ll} \max & F_{\mathrm{CP}}=\sum_{i=0}^{2391}\sum_{\;j=0}^{11}s_{ij}y_{ij}c_{j}\cdot p_{sf}p_{cp} \end{array}\\ \begin{array}{ll} \min & F_{\mathrm{LN}}=\sum_{i=0}^{2391}\sum_{\;j=0}^{11}s_{ij}n_{ij}\varpi_{i}^{\mathrm{LN}} \end{array}\\ \begin{array}{ll} \min & F_{\mathrm{IWU}}=\sum_{i=0}^{2391}\sum_{\;j=0}^{11}s_{ij}w_{ij}\varpi_{i}^{\mathrm{IWU}} \end{array} \end{array}$$

$$\begin{array}{ll} s.t. & \sum_{\;j=0}^{11}s_{ij} \end{array}=S_{i}$$

Here, we have three objective functions: *F*_CP_, *F*_LN_, and *F*_IWU_ to represent national CP, LN, and IWU, respectively. *s_ij_*, *y_ij_*, *n_ij_*, and *w_ij_* are the planting area, crop yield, LN, and IWU of the *j*th CFT over the *i*th county in China, respectively. *c_j_* represents the calories of per unit crop yield (Table S4), and *S_i_* describes the total cropland area within the *i*th county. County-based weights ($\varpi_{i}^{\mathrm{LN}}$ and $\varpi_{i}^{\mathrm{IWU}}$) with the given values of normalized nitrogen surplus and the one minus normalized terrestrial water storage can avoid unrealistic cases where LN increases in highly nutrient-loading regions, and excessive irrigation water demand occurs in arid regions, respectively (see details in Supplementary Note S4). We use the penalty factor (*p_sf_*) to express the constraint about improving self-sufficiency of CP, with the value of minimum self-sufficient rates across five different categories of crop demand (i.e. cereals, soybean, peanut, potato, and rapeseed). Similarly, the penalty factor (*p_cp_*) represents negative effects to the baseline supply chains, with the value of total reduction in CP multiplied by the number of crops with production loss against the baseline levels. We also conducted sensitivity analysis to assess the impacts of the penalty factors on the optimization of crop distributions (Supplementary Note S5), and observed the slight influence (i.e. <4%) on the optimal crop switching under the SSP245 and MRI-ESM-0 (Fig. S12).

In the NSGA-III algorithm, we randomly generated 1,000 initially feasible solutions to construct the population that enables to evolve towards the optimal goals through three major operators (selection, crossover, and mutation). Each solution provides the cover fractions of all CFTs within total 2,391 counties in China. After the 3,000-generation evolution, the NSGA-III algorithm showed a convergence on three optimization objectives (Fig. S13). We then selected all nondominated solutions from the population in the last generation as optimal crop distributions. Consistent with the prediction of future crop demand, our optimization of crop distributions was also conducted at a 5-year step from 2020 to 2100 under each climate scenario. The detailed procedures of NSGA-III algorithm can be found in Supplementary Note S6.

## Agricultural response curves to fertilizer applications

Agricultural response curves (i.e. CP and LN intensity) to fertilization were determined based on the optimal crop distribution maps and different fertilizer levels under unaltered spatial fertilization distributions. We used the climate conditions in the near- (2030), mid- (2050), and long-term (2070) futures when deriving agriculture response to varying fertilization rates. The CP and LN intensity were simulated with total fertilizer applications increasing from 0 Mt to 100 Mt with a step of 5 Mt. Ultimately, the response curves of CP intensity to fertilization were fitted with a logistic function:


$$P(t)=\frac{KP_{0}e^{rt}}{K+P_{0}(e^{rt}-1)}\;$$

where *P*_0_ represents initial CP intensity without fertilization; *K* is the maximum fertilization-induced CP intensity; *r* describes the response rates of CP intensity; and *t* is fertilizer application rates. The response curves of LN intensity to fertilization were fitted based on a polynomial function:


$$\mathrm{LN}(t)=a_{0}t^{3}+a_{1}t^{2}+a_{2}t+a_{3}\;$$

where LN represents the LN intensity in response to fertilization, and *a*_0_-*a*_3_ are polynomial coefficients. All response curves showed satisfactory performance, with the coefficient of determination >0.98 and the relative errors <2% (Table S5).

## Supplementary Material

pgae562_Supplementary_Data

## Data Availability

All five gridded input data are publicly available. The CMFD dataset used in this study is available at https://poles.tpdc.ac.cn/en/data/8028b944-daaa-4511-8769-965612652c49/. Soil property data are available at https://www.isric.org/explore/wise-databases. The CCI-LC dataset is available at https://maps.elie.ucl.ac.be/CCI/viewer/download.php. Global crop-harvested area products are available at http://www.earthstat.org/. National crop survey data are available at https://data.cma.cn. Global nitrogen fertilizer application data are available at https://doi.pangaea.de/10.1594/PANGAEA.863323. Global manure application data are available at https://doi.pangaea.de/10.1594/PANGAEA.871980. In the future projection, the climate data in CMIP6 projects are available at https://esgf-index1.ceda.ac.uk/search/cmip6-ceda/. Future land use data are available at https://data.pnnl.gov/group/nodes/dataset/13192. The GOSIF GPP products are available at https://data.globalecology.unh.edu/data/GOSIF-GPP_v2/. The GLEAM ET products are available at https://www.gleam.eu/. All relevant data generated in this study are available at https://zenodo.org/records/10885828. Source data are provided with this paper. The source codes of LPJ-GUESS model are available at https://zenodo.org/records/8065737. All optimization and display codes are publicly available at https://zenodo.org/records/10885828.
