# Supplementary material for: Improving future agricultural sustainability by optimizing crop distributions in China
Source: PNAS Nexus. 2025 Jan 7;4(1):pgae562. doi: 10.1093/pnasnexus/pgae562 (PMC11705388; doi:10.1093/pnasnexus/pgae562)
Supplement: pgae562_Supplementary_Data [file pgae562_supplementary_data.docx]

**Supplementary Note**

**Note 1: Calculation of crop nitrogen uptake in the LPJ-GUESS model**

In the LPJ-GUESS model, the applications of fertilizer directly enter mineral nitrogen pools in the soils that is accessible for crop uptake. We calculated nitrogen uptake at the daily scale as the lesser between crop demand and the available mineral nitrogen in the soils. Crop nitrogen demand from leaves and roots is associated with the current ratio of C-N because crops require reaching the optimal ratio of C-N in the leaves and roots to maximize the photosynthesis rates. Nitrogen demand from leaves can not only associate with the leaf nitrogen content that optimize the carboxylation capacity (*V*_max_) for photosynthesis, but also expand the crop structural tissues, where daily minimum nitrogen demand (*N_b_*) is calculated based on the associated ratio of C-N in leaves (*C:N_L_*_,max_) and the specific leaf area (*SLA*):

$$N_{b}=\frac{1}{C:N_{L,\max}SLA}$$

Nitrogen demand from roots is proportional to that of leaves because the roots could uptake and transport nutrients to leaves for photosynthesis. The availability of mineral nitrogen for crop uptake depends on soil water content and fine root biomass, as proposed in Prentice (1):

Where *M_N_*_,avail_ represents the availability of mineral nitrogen for crop uptake, describes the projected leaf coverage proportional to the fine root area, *M_N_*_,soil_ is total mass of mineral nitrogen in soils, and $\theta$ is the soil water content.

**Note 2: Determination of CFT’s fractions using an adaptive inverse distance weighting method**

For crops with multiple CFTs, we separated the cover fractions of each CFT from the total cover fractions of the corresponding crop based on national crop surveys and an adaptive inverse distance weighting method (AIDW) (2). In total, we obtained 561 observational stations that provide the information about eleven crop types, their sown and harvest dates from 1992 to 2015 across the entire China (listed in Table S1). In this study, we assumed the fractions of CFTs in each 0.1° grid cell were dependent on two nearest observational stations, where the contributions of these two stations were represented by the inverse-distance weights. We estimated the inverse-distance weights using optimal decay parameters that adaptively vary with spatial distributions of observational stations nationwide. Specifically, the spatial pattern coefficient (*R*) for a given grid cell, representing spatial distributions of observational stations, was calculated as the ratio between actual (*r_obs_*) and expected (*r_exp_*) nearest neighbor distances. We calculated the actual nearest neighbor distance (*r_obs_*) as the average distance between the given grid cell and two nearest observational stations. However, the expected nearest neighbor distance (*r_exp_*) was calculated based on the total number of observational stations (*n*) and total planting area of related crop (*A*).

$$r_{\exp}=\frac{1}{2\times\left(\frac{n}{A}\right)^{0.5}}$$

To derive the optimal decay parameter, the spatial pattern coefficient (*R*) was firstly normalized by min-max normalization method that can be expressed as follows.

$$\mu_{R}=\left\{\begin{matrix} 0 & R<R_{\min} \\ 0.5+0.5\sin\left(\pi/R_{\max}\right)\left(R-R_{\min}\right) & R_{\min}\leq R\leq R_{\max} \\ 1 & R>R_{\max} \end{matrix}\right.$$

Where *R*_min_ and *R*_max_ are empirical minimum and maximum values of spatial pattern coefficient, with the default values of 0.2 and 3.5, respectively. The optimal decay parameters ($\alpha$) were then determined with the normalized spatial pattern coefficient ($\mu_{R}$) and their standard triangular relationship (see Fig. 3 in refs (2)). We also calculated the inverse-distance weights by an exponential function with optimal decay parameters. Finally, the cover fraction of each CFT was calculated as the fractions of associated crop multiplied by the inverse-distance weights.

**Note 3: Projection of nitrogen fertilizer and manure application under future climate change**

We projected nitrogen fertilizer and manure applications based on the prediction of total synthetic fertilizer (i.e., nitrogen fertilizer and compound fertilizer, Table S6) and animal numbers (Table S7) at the national scale in 2010, 2030 and 2050 across the five different scenarios in refs (3). We assumed that nitrogen content of synthetic fertilizer was 46.7% and 13.3%, respectively, which were then used to nitrogen applications from synthetic fertilizer. As for nitrogen applications from manure, we collected the relevant information, such as the feeding periods and manure nitrogen coefficients for each animal (Table S8) from refs (4, 5), and then calculated using the following equation:

$$TN_{m}=\sum_{i=1}^{n} N_{i}\cdot FP_{i}\cdot MNC_{i}$$

Where *N_i_* is the total number of the *i*-th raised animal, *FP_i_* and *MNC_i_* are the associated feeding days and manure nitrogen coefficients. Ultimately, we determined the relative changes in total nitrogen applications from synthetic fertilizer and manure from 2010 to 2050 under three different SSPs. As a result, the predicted nitrogen applications from fertilizer and manure decrease by 5%, 20% and 6% from 2010 to 2030, and by 59%, 14% and 9% from 2030 to 2050 under SSP126, SSP245, and SSP585, respectively.

**Note 4: County-based weights for leached nitrogen and irrigation water use**

In the optimization of crop distributions, we employed county-based weights to avoid unrealistic crop-switching schemes, such as the large increase in leached nitrogen over high-level nitrogen-loading regions, and the greater irrigation water demand in arid regions. Nitrogen surplus, as the LPJ-GUESS simulated difference between total nitrogen inputs and the harvested nitrogen from 1979 to 2014, was used here to represent the historical levels of nitrogen loadings in soils. We normalized the averaged nitrogen surplus using the min-max normalization method in Python and regarded their values as the leached-nitrogen weights. We used a global terrestrial water storage dataset that is simulated by WaterGAPs model (version 2.2d) and has a spatial resolution of 0.5° (6) to evaluate water availability in China from 1979 to 2014. In this dataset, the Earth’s surface and groundwater are included to represent water resources available for irrigation and human consumption. It was then clipped and calculated the averaged values of terrestrial water storage within each county to describe irrigation water availability. We employed the same min-max method to normalize terrestrial water storage and subtracted them from the one as irrigation-water-use weights. It is noted that we assumed that the county-based weights for water availability were unaltered under future climate change.

**Note 5: Sensitivity analysis of the penalty factor in the optimization**

We conducted the sensitivity analysis to evaluate the impacts of the penalty factors in the multi-objective optimization on crop switching. All penalty factors about the constraints of improving self-sufficiency and of minimizing the negative effects to the supply chains were normalized using the Z-score method and then scaled into the range between -10 and 10 by multiplying 10 to run the optimization crop distributions. We then selected the moderate-emission scenario (i.e., SSP245 projected by MRI-ESM2-0) as the representative one to optimize crop distributions with the normalized penalty factors because crop switching is nearly uniform across the different GCMs and SSPs. The impacts of the penalty factors were ultimately assessed by comparing the optimal CFT fractions.

**Note 6: The specific procedures of the NSGA-III algorithm**

The Non-dominated Sorting Genetic Algorithm III (III) proposed by refs (7) is an evolutionary many-objective optimization algorithm that enables to solve the problems with three or more conflicting objectives. It has been widely used to generate robust and practicable solutions in many fields, such as engine calibration (8, 9), software optimization (10, 11), and parameter estimation (12, 13). Compared with the traditional evolutionary algorithm yielding one optimal solution for single-objective problems, the NSGA-III algorithm can generate a set of “Pareto optimal solutions” (also called “non-dominated solutions”) that reflects the trade-offs of solutions among different optimization objectives. Pareto optimal solutions are those with any of the objective values that cannot be improved without the degradations in other objectives. In the NSGA-III algorithm, Pareto optimal solutions are distinguished from the population based on the Pareto dominance relation.

As common procedures of evolutionary algorithms, the NSGA-III algorithm also starts with randomly generating initial population with the pre-defined number (*N*) of individuals, where each individual defines a feasible solution to optimization problems. Initial population is then updated through selection, crossover, and mutation operators in the evolutionary process. After the predefined-generation evolution, all non-dominated individuals are obtained as the globally optimal solutions. Specifically, all individuals in the population are firstly separated into two classes as parents to generate offspring. Each pair of parent individuals ideally generate two offspring through crossover and mutation operators. There are various existing crossover and mutation methods, such as single-point crossover, uniform crossover, simulated binary crossover, inversion mutation, scramble mutation, and polynomial mutation (14, 15). In this study, we used the simulated binary crossover method that can be expressed as follows:

$$\left\{\begin{matrix} c_{i}^{1}=0.5\times\left[\left(1+\beta\right)\cdot x_{i}^{1}+\left(1-\beta\right)\cdot x_{i}^{2}\right] \\ c_{i}^{2}=0.5\times\left[\left(1-\beta\right)\cdot x_{i}^{1}+\left(1+\beta\right)\cdot x_{i}^{2}\right] \end{matrix}\right.$$

$$\beta=\left\{\begin{matrix} \left(rand\times2\right)^{1/\left(1+\eta\right)} & rand\leq0.5 \\ \left[1/\left(2-rand\times2\right)\right]^{1/\left(1+\eta\right)} & rand>0.5 \end{matrix}\right.$$

Two parent individuals are assumed as $X^{1}\left(x_{1}^{1},x_{1}^{1},...,x_{n}^{1}\right)$ and $X^{2}\left(x_{1}^{2},x_{1}^{2},...,x_{n}^{2}\right)$, respectively; Two generated offspring individuals are assumed as $C^{1}\left(c_{1}^{1},c_{1}^{1},...,c_{n}^{1}\right)$ and $C^{2}\left(c_{1}^{2},c_{1}^{2},...,c_{n}^{2}\right)$; *rand* is the random number; and $\eta$ represents a predefined parameter, with the value of 20, where the higher value of $\eta$ means the more similarity between offspring and parent individuals. Mutation operator can effectively maintain the individual diversity of the population and thus avoid the evolution trapped into local optimal solutions. The polynomial mutation method is used here and expressed as follows:

$$\upsilon'_{k}=\upsilon_{k}+\delta\cdot\left(u_{k}-l_{k}\right)$$

$$\delta=\left\{\begin{matrix} \left[2u+\left(1-2u\right)\left(1-\delta_{1}\right)^{\eta_{m}+1}\right]^{\frac{1}{\eta_{m}+1}} & u\leq0.5 \\ 1-\left[2\left(1-u\right)+2\left(u-0.5\right)\left(1-\delta_{2}\right)^{\eta_{m}+1}\right]^{\frac{1}{\eta_{m}+1}} & u>0.5 \end{matrix}\right.$$

$$\left\{\begin{matrix} \delta_{1}=\left(\upsilon_{k}-l_{k}\right)/\left(u_{k}-l_{k}\right) \\ \delta_{2}=\left(u_{k}-\upsilon_{k}\right)/\left(u_{k}-l_{k}\right) \end{matrix}\right.$$

Where $\upsilon_{k}$ represents the individual prior to mutation operator; $u_{k}$ and $l_{k}$ are the upper and lower bounds of individuals, respectively; *u* is the random number; and $\eta$ is the pre-defined distribution index, with the value of 20. All parent and offspring individuals are candidates for the population in next generation that is constructed with the best individuals (total number as *M*) determined through selection operator. The selection operator comprises five major steps briefly described as follows:

1. *Classification of all individuals into different non-dominated levels*

We used the usual dominance principle to identify the non-dominated levels of each individual that are assumed as *F*_1_, *F*_2_, and so on (16). All individuals are then sorted by their non-dominated levels. We select individuals in each non-dominated level to construct a new population (*S_t_*) that covers the levels from *F*_1_ to *F_l_* when its size is equal or greater than *N* for the first time. Individuals in the last level *F_l_* might be partially selected if the size of *S_t_* exceeds *N*. In such cases, we should select the individuals that can maintain the diversity of the last level.

1. *Generation of pre-defined reference points*

However, the increasing number of optimization objectives might result in the failure of selection with maximum diversity. The NSGA-III algorithm introduces a set of reference points predefined by the Das and Dennis’s systematic approach to solve this issue (17). The reference points are required to widely distribute on a normalized hyper-plane, and their number depends on the number of objectives and the division on each objective axis. For example, three-objective problems with four divisions on each objective axis generally require 15 reference points.

1. *Normalization of all individuals*

All individuals in the population *S_t_* are adaptively normalized with the ideal point and extreme points. The ideal point represents the minimum value ($z_{i}^{\min}$) for each objective in the population *S_t_*. Each objective value (*f_i_*) for each individual is then subtracted by its associated ideal value, which is assumed as the translated objective value $f_{i}^{'}$ . Thereafter, the extreme points are determined as the individuals that make the corresponding achievement scalarizing the minimum value on each objective axis. Note that the number of extreme points is equal to that of optimization objectives (*M*). Using these extreme points can generate a hyper-plane, and then calculate the intercept (*a*) of each objective axis and this hyper-plane. Hence, the objective values can be normalized as:

$$\begin{matrix} f_{i}^{n}=\frac{f_{i}^{'}}{a_{i}}, & i=1,2,...,M \end{matrix}$$

Notably, the predefined reference points already lie on the normalized hyper-plane constructed with the intercepts of each objective axis.

1. *Association of reference points with individuals*

After adaptive normalization of individuals, each individual are associated with a reference point. Firstly, a reference line between each reference point and the origin is defined to determine its perpendicular distance to all individuals. The individual is considered to associate with the reference points when it shows the minimum distance to the corresponding reference line, meaning that a reference point may associate with one or more individuals.

1. *Niche-preservation operation*

Finally, we select totally *N* individuals from the population *S_t_* as the population in next generation (*P_n_*). All individuals in the levels *F*_1_-*F_l_*_-1_ (assumed total number of individuals as *N_D_*) are directly added into next generation. The rest individuals are required to select from the last level *F_l_* to ensure the size of population in next generation as *N*. Firstly, we select the reference points with the smallest number of associated individuals. If such reference points are more than one, we randomly choose one reference point. From its associated individuals, the individual in the last level and holding the smallest perpendicular distance to the reference line is one population member of next generation. Simultaneously, the number of associated individuals is also subtracted by one. If this reference point already has an associated individual added into next generation, we randomly select one from the other associated individuals in the last level into next generation. Similarly, the number of associated individuals is further subtracted by one. Such selection strategies are repeated until the population size in next generation as *N*.

**Supplementary Tables**

**Table S1 |** **Crops and associated CFTs in our simulations.** The parameter values applied for the calibrated LPJ-GUESS model are also presented here, where the parameters include specific leaf area (SLA), sowing temperature limits (STL), and default length of growing seasons (LGS).

| Crops | Rotations | CFTs | Hydrology | SLA | STL | LGS |
| --- | --- | --- | --- | --- | --- | --- |
| Early-season rice | Yes | TrER | Irrigated | 50 | 135 | 15 |
| Late-season rice | Yes | TrLR | Irrigated | 60 | 135 | 15 |
| Single-season rice | No | TrSR | Irrigated | 50 | 230 | 18 |
| Spring wheat | No | TeSW | Irrigated | 35 | 160 | 13 |
| Winter wheat | No | TeWW | Irrigated | 35 | 250 | 13 |
| Spring maize | No | TeCoSp | Irrigated | 30 | 140 | 14 |
| Summer maize | Yes | TeCoSu | Irrigated | 45 | 90 | 14 |
| Soybean | No | TeSo | Irrigated | 30 | 110 | 13 |
| Rapeseed | No | TeRa | Irrigated | 30 | 230 | 17 |
| Peanut | No | TrPe | Irrigated | 45 | 120 | 15 |
| Potato | No | TrMa | Rain-fed | 45 | 120 | 22 |

**Table S2 | Land cover types clustered into cropland for historical and future periods.**

|  | Land cover types | Type Index | Weights |
| --- | --- | --- | --- |
| ESA-CCI | Rain-fed cropland | 10 | 1.0 |
|  | Irrigated cropland | 20 | 1.0 |
|  | Mosaic cropland | 30 | 0.5 |
| GCAM-LU | Corn: rain-fed | 15 | 1.0 |
|  | Corn: irrigated | 16 | 1.0 |
|  | Wheat: rain-fed | 17 | 1.0 |
|  | Wheat: irrigated | 18 | 1.0 |
|  | Soybean: rain-fed | 19 | 1.0 |
|  | Soybean: irrigated | 20 | 1.0 |
|  | Rice: rain-fed | 23 | 1.0 |
|  | Rice: irrigated | 24 | 1.0 |
|  | Sugar crop: irrigated | 25 | 1.0 |
|  | Sugar crop: rain-fed | 26 | 1.0 |
|  | Other crop: irrigated | 27 | 1.0 |
|  | Other crop: rain-fed | 28 | 1.0 |
|  | Bioenergy crop: rain-fed | 29 | 0.5 |
|  | Bioenergy crop: irrigated | 30 | 0.5 |

**Table S3 | Feed-meat conversion rates for animal calorie demand in China (18, 19).** Note that we regarded barley into cereals in this study.

|  | Wheat | Maize | Soybean | Rapeseed | Nuts | Barley | Roots |
| --- | --- | --- | --- | --- | --- | --- | --- |
| Dairy and eggs | 0.61 | 0.61 | 1.31 | 0.28 | 0 | 0.03 | 0 |
| Ruminant meat | 0.48 | 0.43 | 0.77 | 1.10 | 1.03 | 0.66 | 0 |
| Poultry | 0.22 | 0.43 | 1.27 | 0.25 | 0 | 0 | 0 |
| Pork | 0.68 | 0.39 | 1.11 | 0.25 | 0.25 | 0.27 | 0.01 |
| Seafood | 0.48 | 1.31 | 1.71 | 0 | 0 | 0 | 0 |

**Table S4 | FAOSTAT conversion values of crop calories used in our study.**

| Crops | rice | maize | wheat | soybean | peanut | potato | rapeseed |
| --- | --- | --- | --- | --- | --- | --- | --- |
| kcal kg^-1^ | 3600 | 3560 | 3340 | 3350 | 5890 | 670 | 4940 |

**Table S5 | Overall performance of agricultural response curves to fertilizer applications.** Here, statistical indicators include the coefficient of determination (R^2^), mean relative error (MRE), and root mean squared error (RMSE).

|  | | Crop production | | | | Leached nitrogen | | |  |
| --- | --- | --- | --- | --- | --- | --- | --- | --- | --- |
|  |  | R^2^ | MRE/% | RMSE/% | R^2^ | | MRE/% | RMSE/% | |
| SSP126 | 2030 | 0.99 | 1.37 | 2.33 | 1.00 | | 2.63 | 4.76 | |
|  | 2050 | 0.99 | 1.82 | 2.81 | 1.00 | | 3.15 | 5.94 | |
|  | 2070 | 0.99 | 1.89 | 2.73 | 1.00 | | 2.66 | 4.77 | |
| SSP245 | 2030 | 0.99 | 1.22 | 2.05 | 1.00 | | 2.21 | 5.94 | |
|  | 2050 | 0.99 | 1.41 | 2.33 | 1.00 | | 1.47 | 2.82 | |
|  | 2070 | 0.99 | 1.86 | 3.04 | 1.00 | | 1.50 | 2.69 | |
| SSP585 | 2030 | 0.99 | 1.18 | 2.17 | 1.00 | | 3.30 | 10.65 | |
|  | 2050 | 0.99 | 1.65 | 2.98 | 1.00 | | 2.13 | 4.69 | |
|  | 2070 | 0.98 | 1.94 | 3.40 | 1.00 | | 1.53 | 2.70 | |

**Table S6 | Projected applications of synthetic fertilizer (10^3^ ton) in China using the NUFER model in 2010, 2030 and 2050.**

|  | 2010 | 2030 | | | 2050 | | |
| --- | --- | --- | --- | --- | --- | --- | --- |
|  |  | SSP1 | SSP2 | SSP5 | SSP1 | SSP2 | SSP5 |
| Nitrogen fertilizer | 23537 | 2354 | 14122 | 18830 | 0 | 12004 | 16947 |
| Compound fertilizer | 17985 | 1799 | 10791 | 14388 | 0 | 9172 | 12949 |

**Table S7 | Model-predicted animal numbers (10^6^ head yr^-1^) in China in 2010, 2030 and 2050.**

|  | 2010 | 2030 | | | 2050 | | |
| --- | --- | --- | --- | --- | --- | --- | --- |
|  |  | SSP1 | SSP2 | SSP5 | SSP1 | SSP2 | SSP5 |
| Poultry | 10609 | 13792 | 15914 | 16975 | 12413 | 14323 | 15278 |
| Mule and donkey | 9 | 12 | 14 | 15 | 11 | 12 | 13 |
| Horse | 7 | 9 | 10 | 11 | 8 | 9 | 10 |
| Milk cow | 13 | 16 | 19 | 20 | 15 | 17 | 18 |
| Meat cow | 47 | 61 | 71 | 75 | 55 | 64 | 68 |
| Rabbit | 433 | 563 | 649 | 693 | 506 | 584 | 623 |
| Sheep and goat | 281 | 365 | 421 | 449 | 329 | 379 | 404 |
| Other cows | 43 | 55 | 64 | 68 | 50 | 58 | 61 |
| Pig | 667 | 867 | 1000 | 1067 | 780 | 900 | 960 |

**Table S8 | Information about the feeding periods (FP, days) and manure nitrogen coefficient (MNC, g N day^-1^) for each animal in China**

| Animals | FP | MNC | Animals | FP | MNC |
| --- | --- | --- | --- | --- | --- |
| Poultry | 210 | 1.20 | Rabbit | 90 | 1.16 |
| Mule and donkey | 365 | 12.4 | Sheep and goat | 365 | 2.15 |
| Horse | 365 | 12.4 | Other cows | 365 | 115.99 |
| Milk cow | 365 | 250.04 | Pig | 199 | 30.35 |
| Meat cow | 365 | 108.53 |  |  |  |

**Supplementary Figures**

**
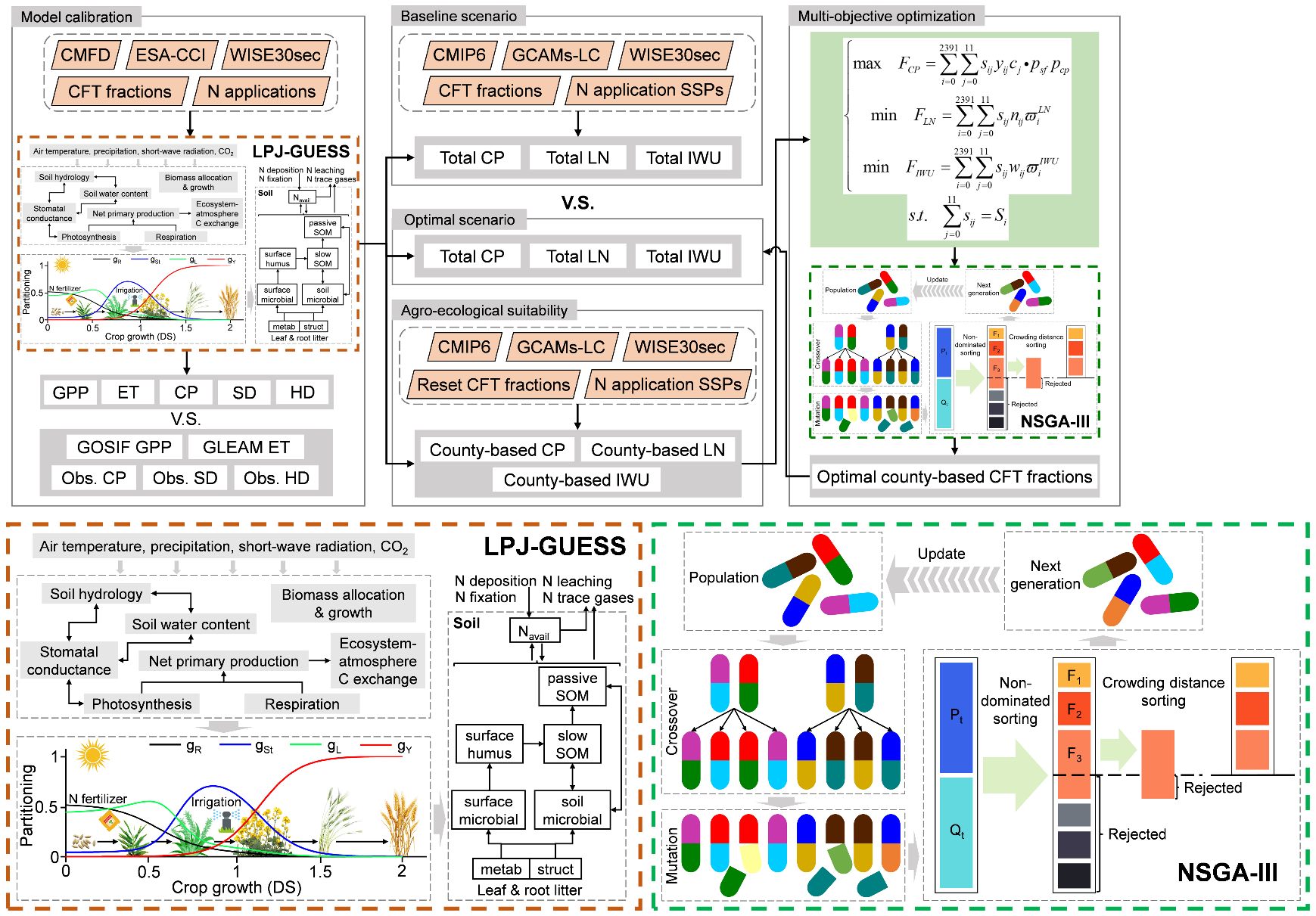
**

**Fig. S1 | Procedures of optimizing crop distributions in future China’s agriculture ecosystems.** Here we divided the whole process of optimizing crop distributions at the national scale into four main procedures: 1) Model calibration: the LPJ-GUESS model was calibrated and evaluated based on in-situ dataset for the historical period (1979-2014). 2) Mapping agro-ecological suitability: the calibrated LPJ-GUESS model driven by the reset CFT fractions were used to simulate crop yield, leached nitrogen and irrigation water use as the agro-ecological suitability under the different SSPs. 3) Multi-objective optimization: crop distributions were optimized spatially with three conflicting objectives, including maximum of crop production, minimum of leached nitrogen, and minimum of irrigation water use at the national scale. 4) Benefit assessments: the overall benefits were assessed by comparing total crop production, leached nitrogen and irrigation water use between baseline and optimal scenarios.

**
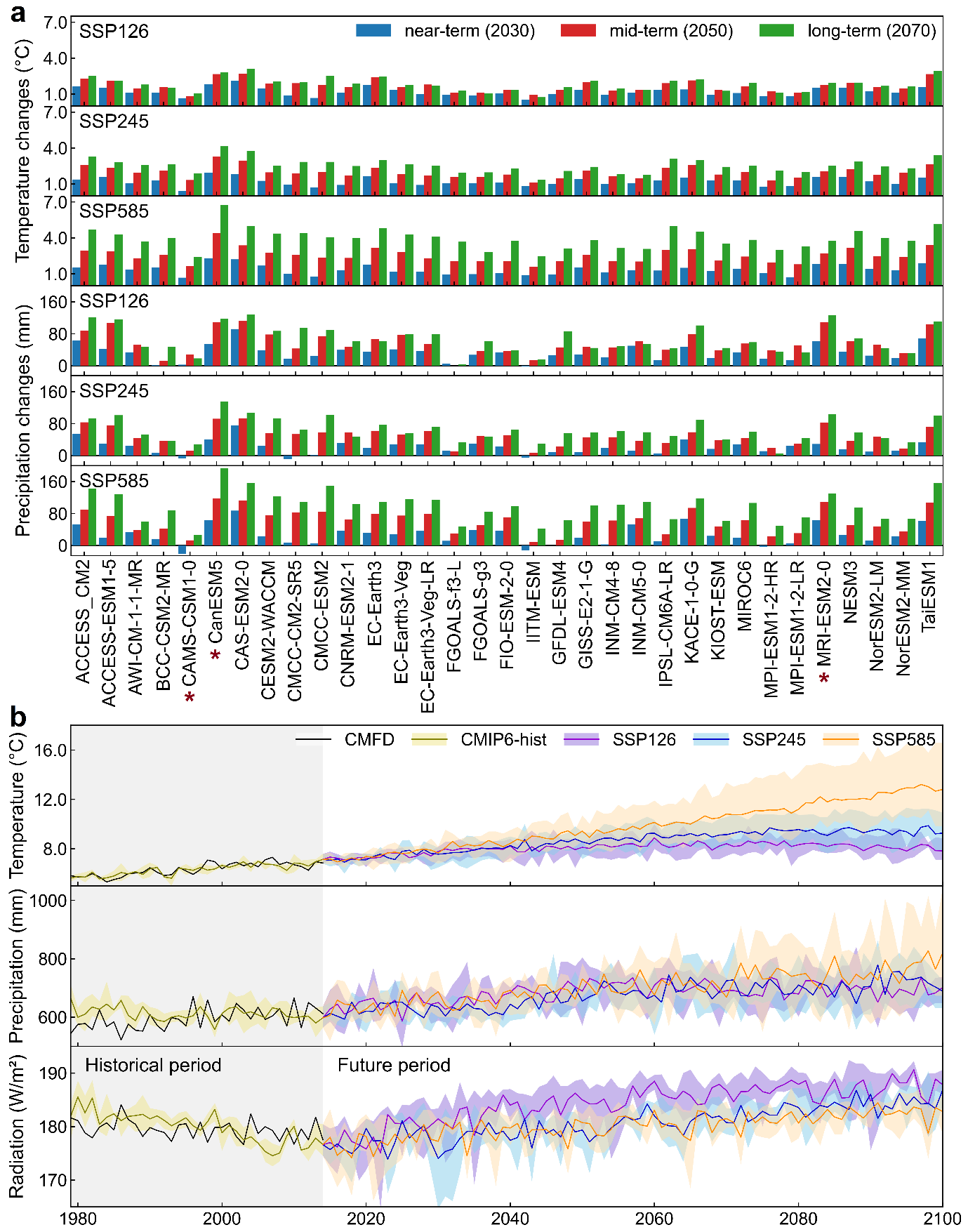
**

**Fig. S2 | Selection and bias-correction of future climate scenarios in the CMIP6 project.** (**a**) Comparison of temperature and precipitation changes projected by the accessible general circulation models (GCMs) in the near- (2021-2040), mid- (2041-2060) and long-term futures (2061-2080) in CMIP6 project. All temperature and precipitation changes were calculated as the difference against the historical period (1985-2014). The selected GCMs are marked as ‘*’ to represent the low-, moderate-, and high-level climate changes in the future periods. (**b**) Bias-corrected climate data in the future period, where comparing the CMFD and bias-corrected data generated by GCMs shows the satisfactory performance of bias-correction. The colored shadows show the climate ranges of different GCMs.

**
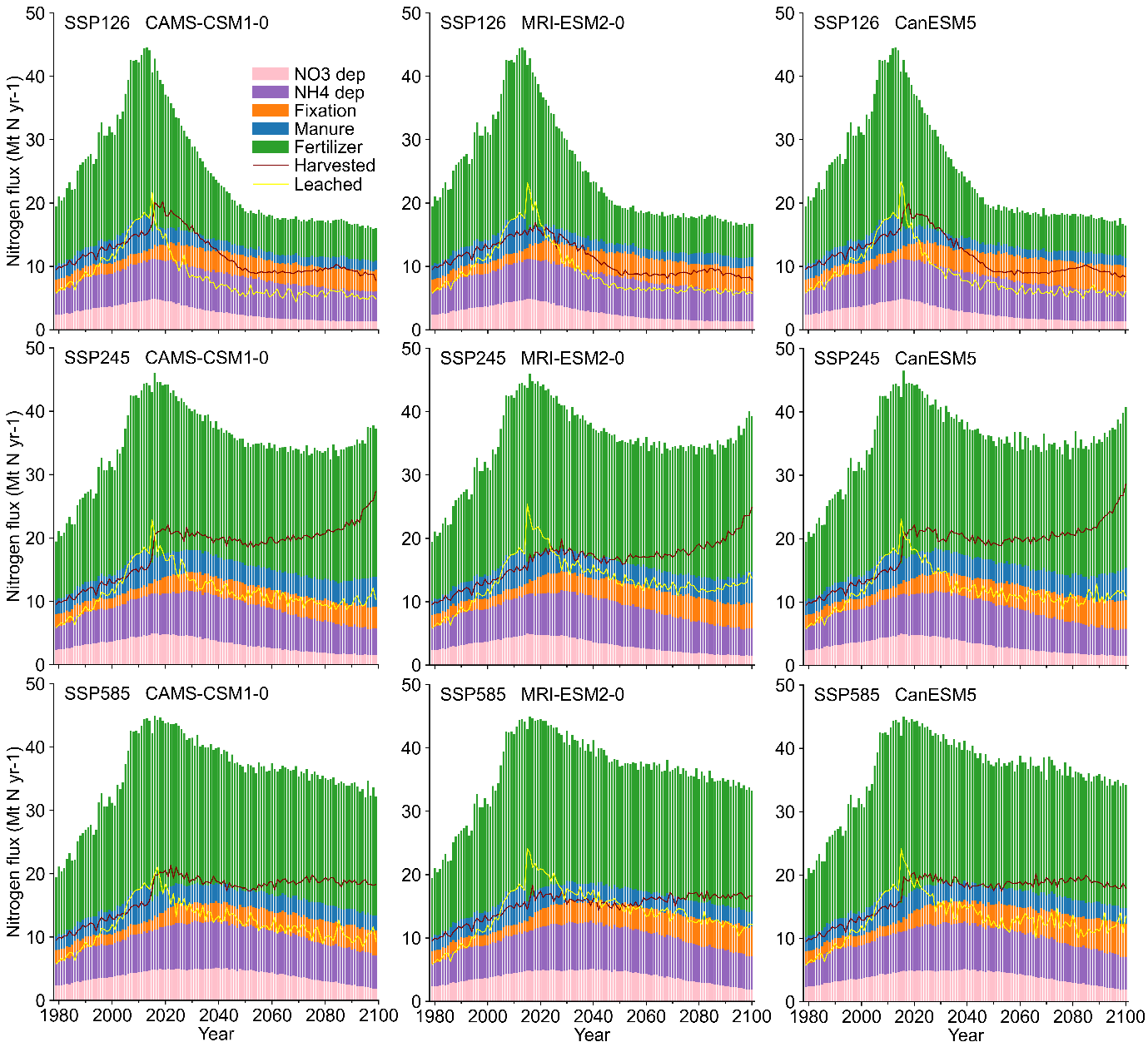
**

**Fig. S3 | Overall nitrogen fluxes in China’s agriculture ecosystems for the historical (1979-2014) and future (2015-2100) periods.** All five accessible nitrogen sources for agriculture ecosystems, including atmospheric deposition, biological fixation, and chemical fertilizer and manure applications, are color-coded boxes, while the colored lines of harvested and leached nitrogen represent agriculture outputs under each GCM and SSP.


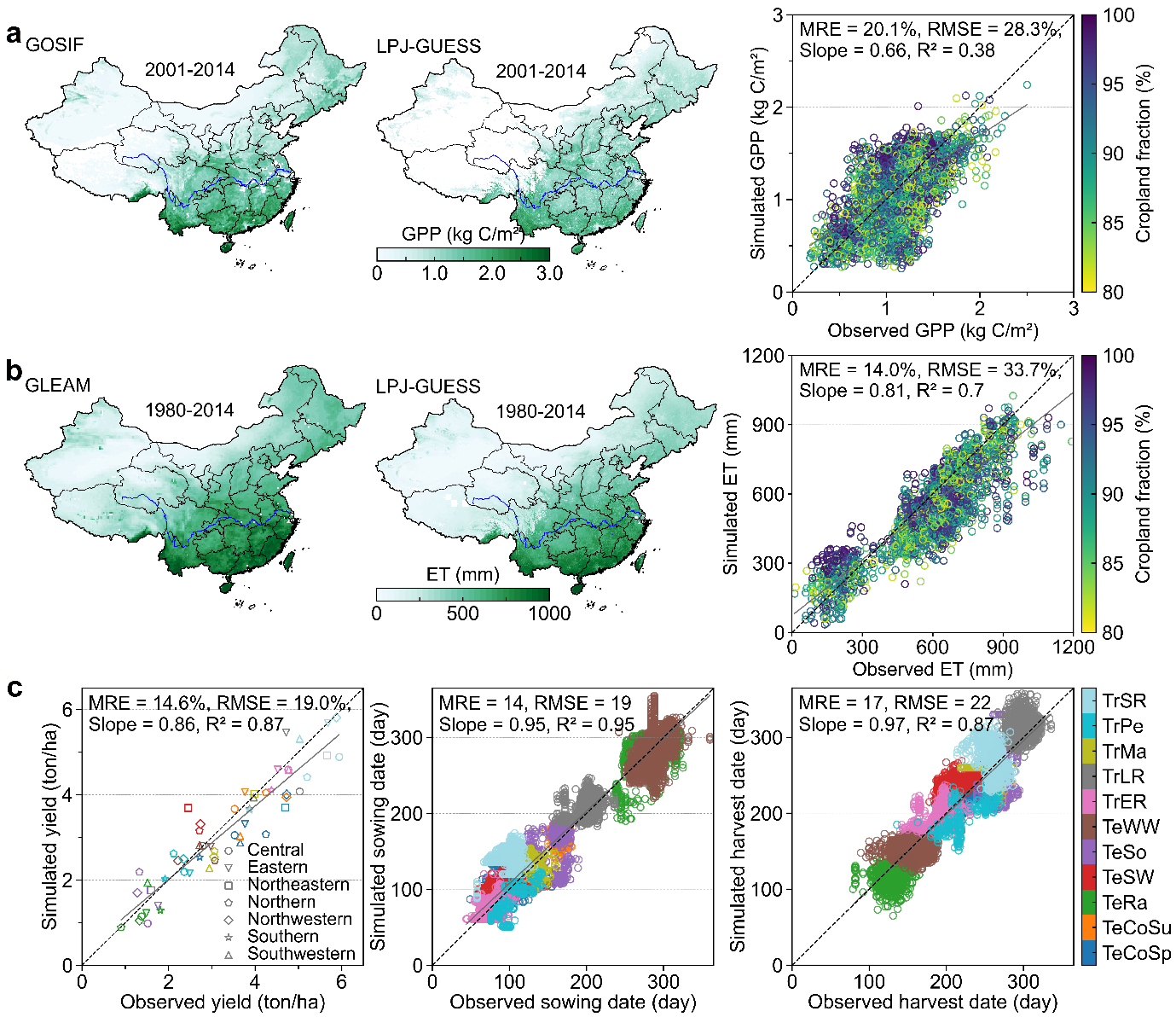


**Fig. S4** **| Evaluation of the LPJ-GUESS simulations for the historical period.** Spatial distributions of climatological evaluated and simulated gross primary production (GPP, **a**), and evapotranspiration (ET, **b**) across the entire China, where the scatterplots in the right panels show the comparisons on cropland-dominant (cropland fraction > 80%) grid cells. (**c**) Evaluation of simulated crop yield, sowing and harvest dates for different crops, where the colors and shapes of scatter points represent crop and region types in China.


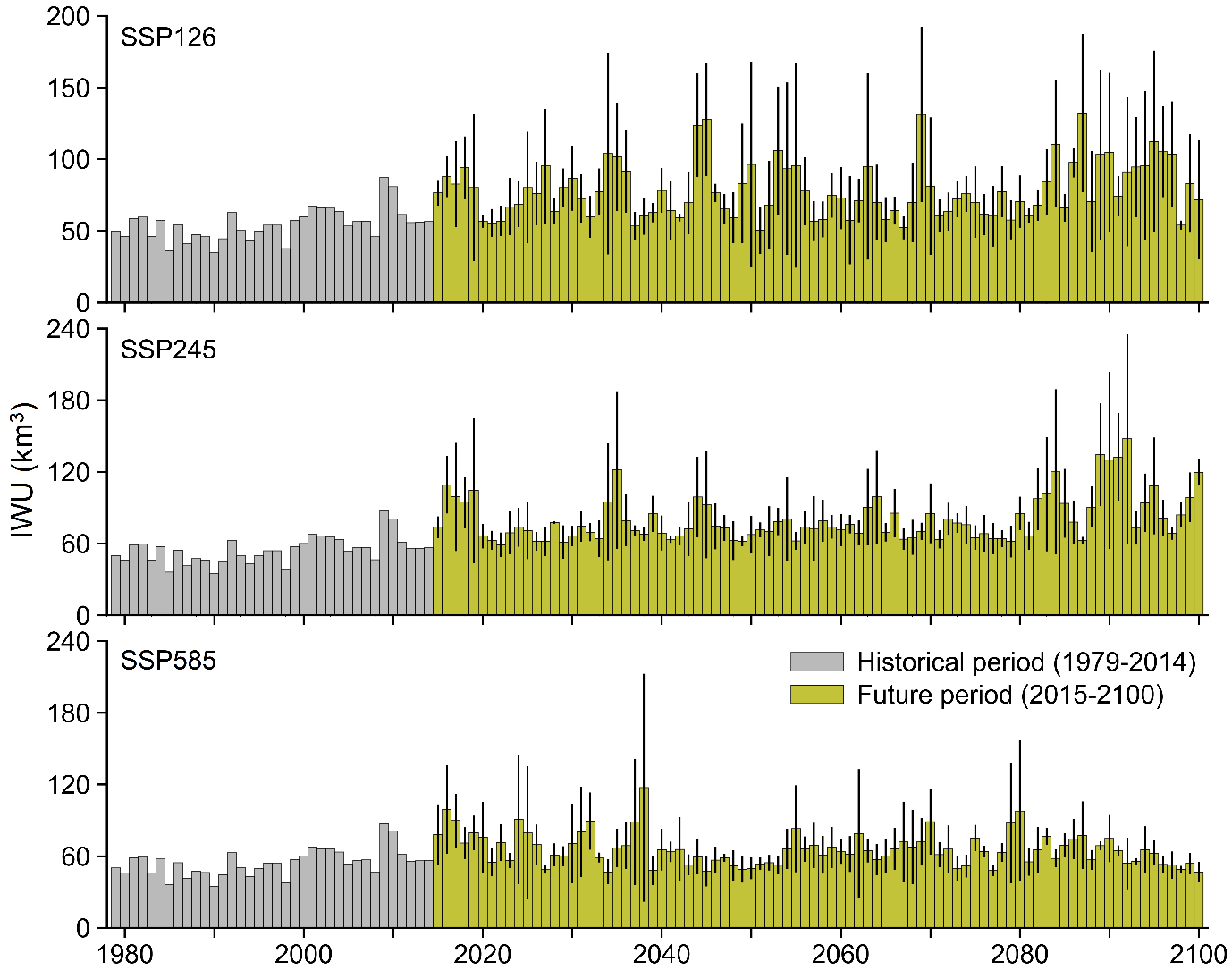


**Fig. S5 | Temporal dynamics of irrigation water use (IWU) in agriculture ecosystems.** The error bars show one standard deviation among three GCMs under each SSP.


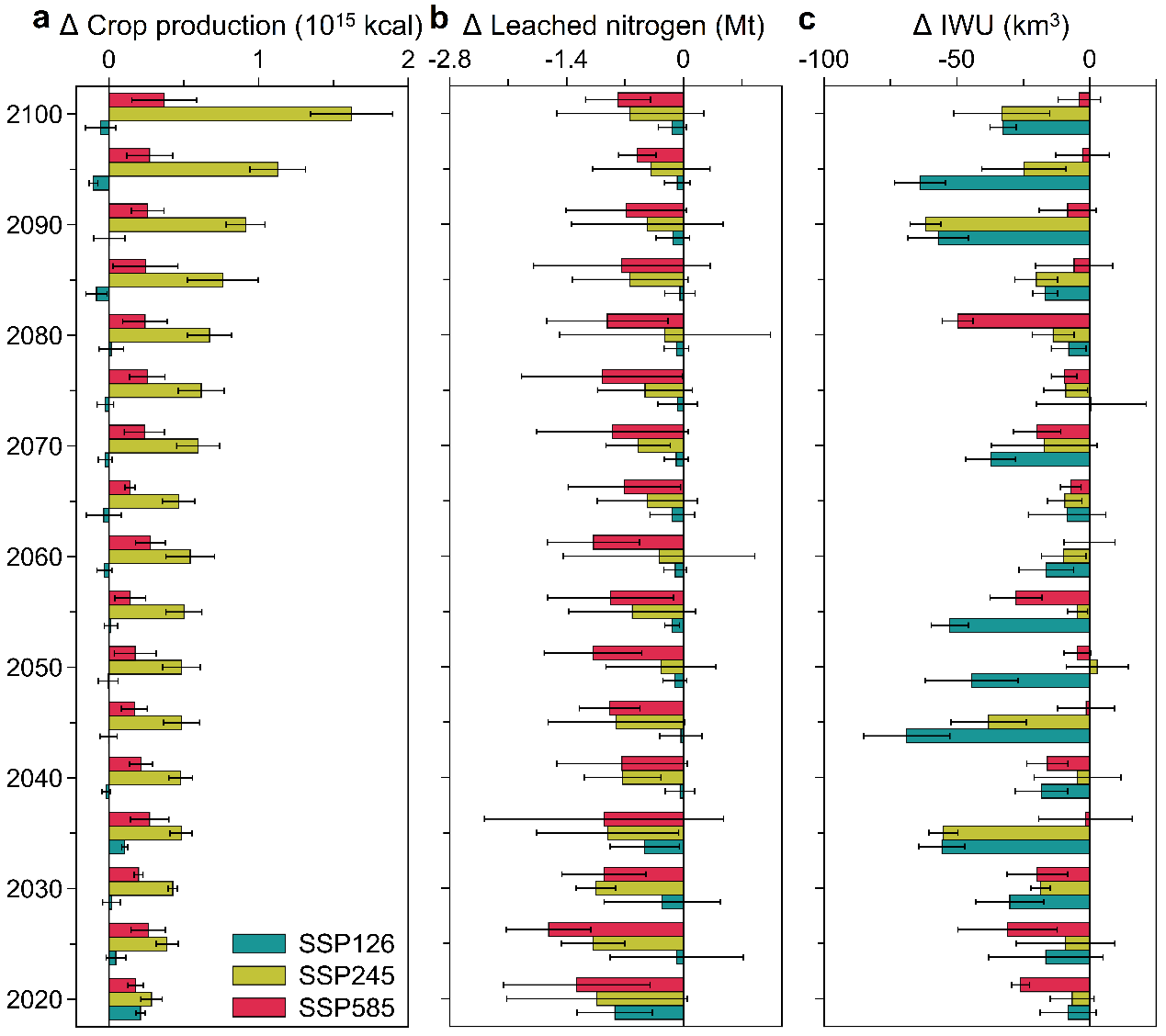


**Fig. S6 | Optimization-induced absolute changes in crop production (CP), leached nitrogen (LN), and irrigation water use (IWU) in the future period.** The color-coded boxes represent three different SSPs, and the error bars show one standard deviation among three GCMs, consistent to Fig. 2.

**
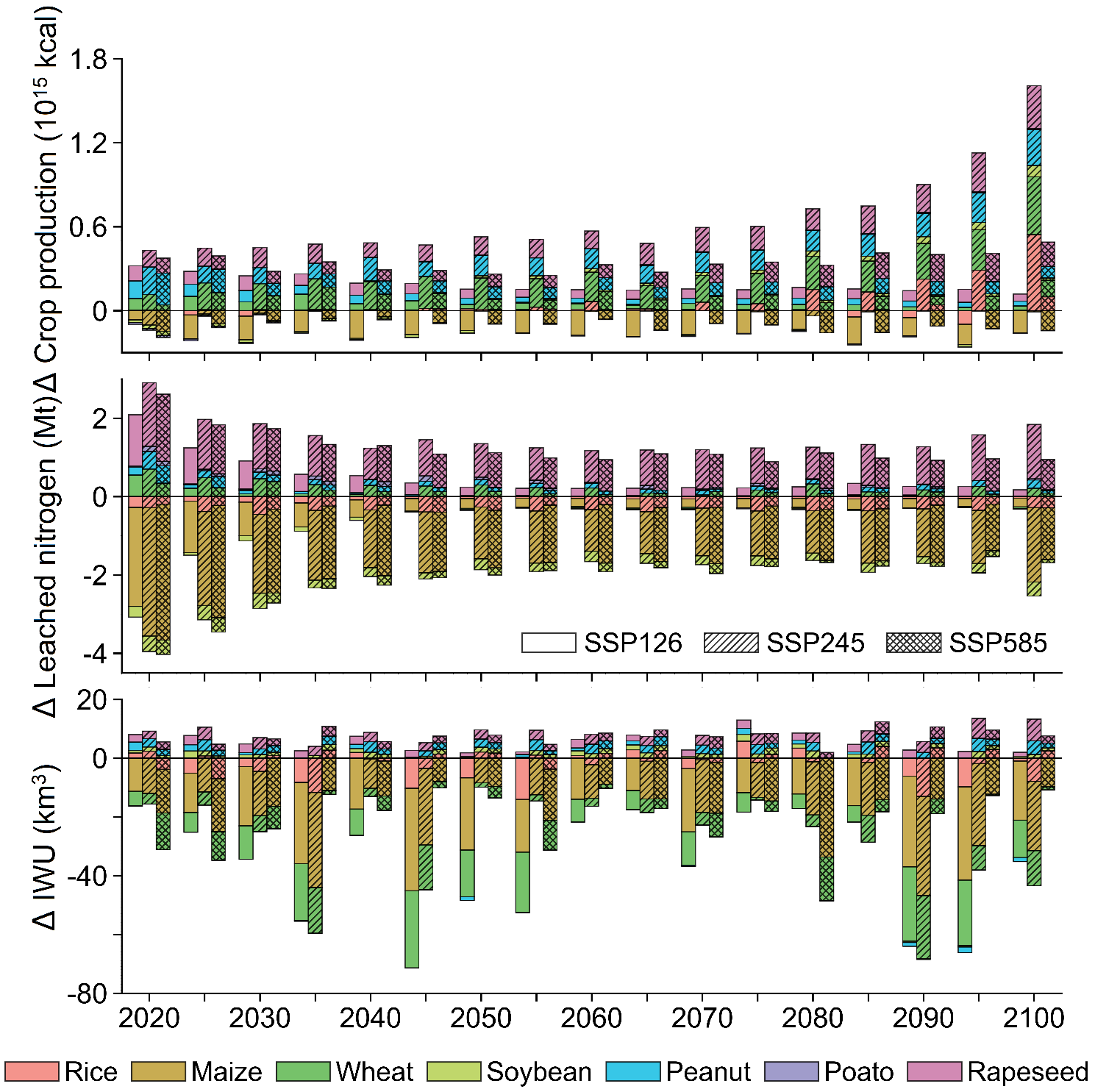
**

**Fig. S7 | Optimization-induced benefits contributed by different crops in the future climate and fertilization scenarios.** As illustrated in Fig. 2, color-coded crop-specific optimization benefits were calculated as the difference between optimal and baseline levels. The upper, central and lower panels show the benefits on crop production, leached nitrogen and irrigation water use (IWU), respectively, for the future period. For total benefits on these three indicators, please refer to Fig. 2 and Fig. S5.

**
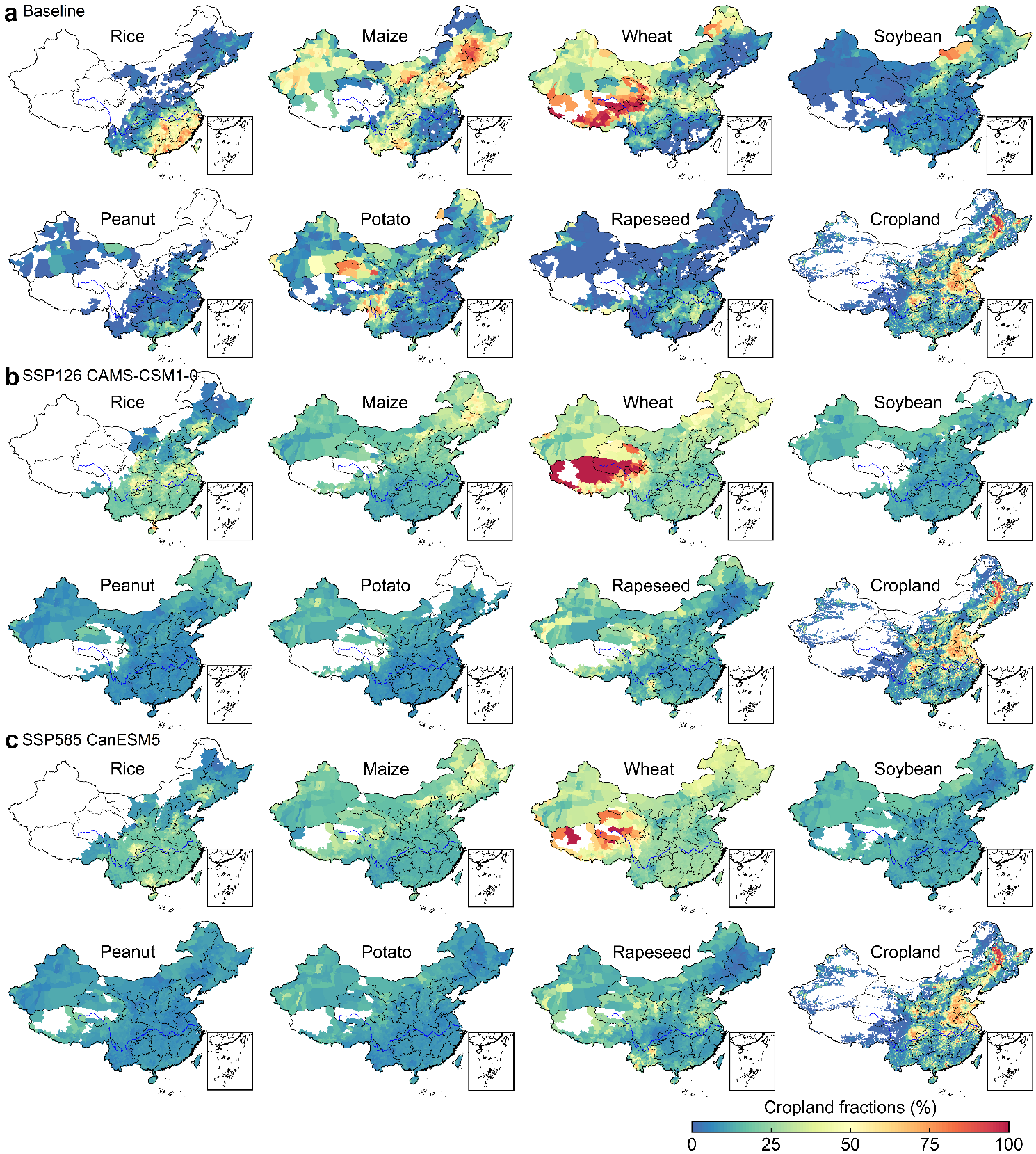
**

**Fig. S8 | Examples showing spatial discrepancies in crop distributions between the baseline and optimal scenarios.** (**a**) The baseline scenario was assumed to be consistent with the historical patterns that was determined with global crop harvested area maps and field-survey datasets (see Methods). Notably, the baseline crop distributions are homogeneous among all three GCMs and SSPs. (**b**) and (**c**) The optimal crop distribution maps represented by the lowest- and highest-level climate change show the average patterns of all non-dominated solutions generated by the NSGA-III algorithm for the period of 2020-2100 (see Methods).


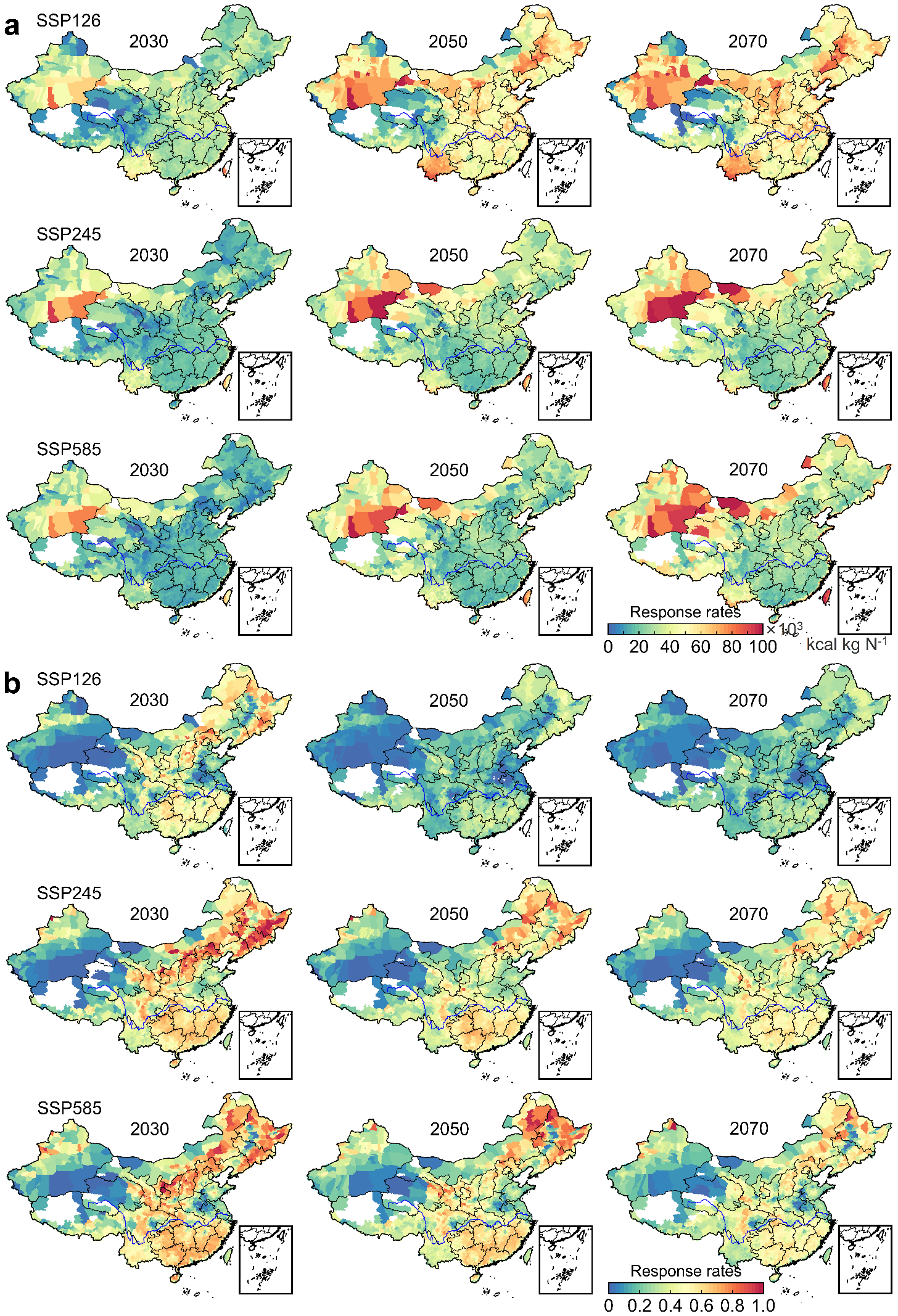


**Fig. S9 | Spatial variations in agriculture response to the baseline fertilization management.** (**a**) and (**b**) County-based response rates of crop production and leached nitrogen for the near- (2030), moderate- (2050), and long-term (2070) futures of each SSP: SSP126 (top panels), SSP245 (central panels) and SSP585 (bottom panels), respectively. The units of response rates demonstrate the potential increases in crop production and leached nitrogen when enhancing one unit of fertilizer applications.

**
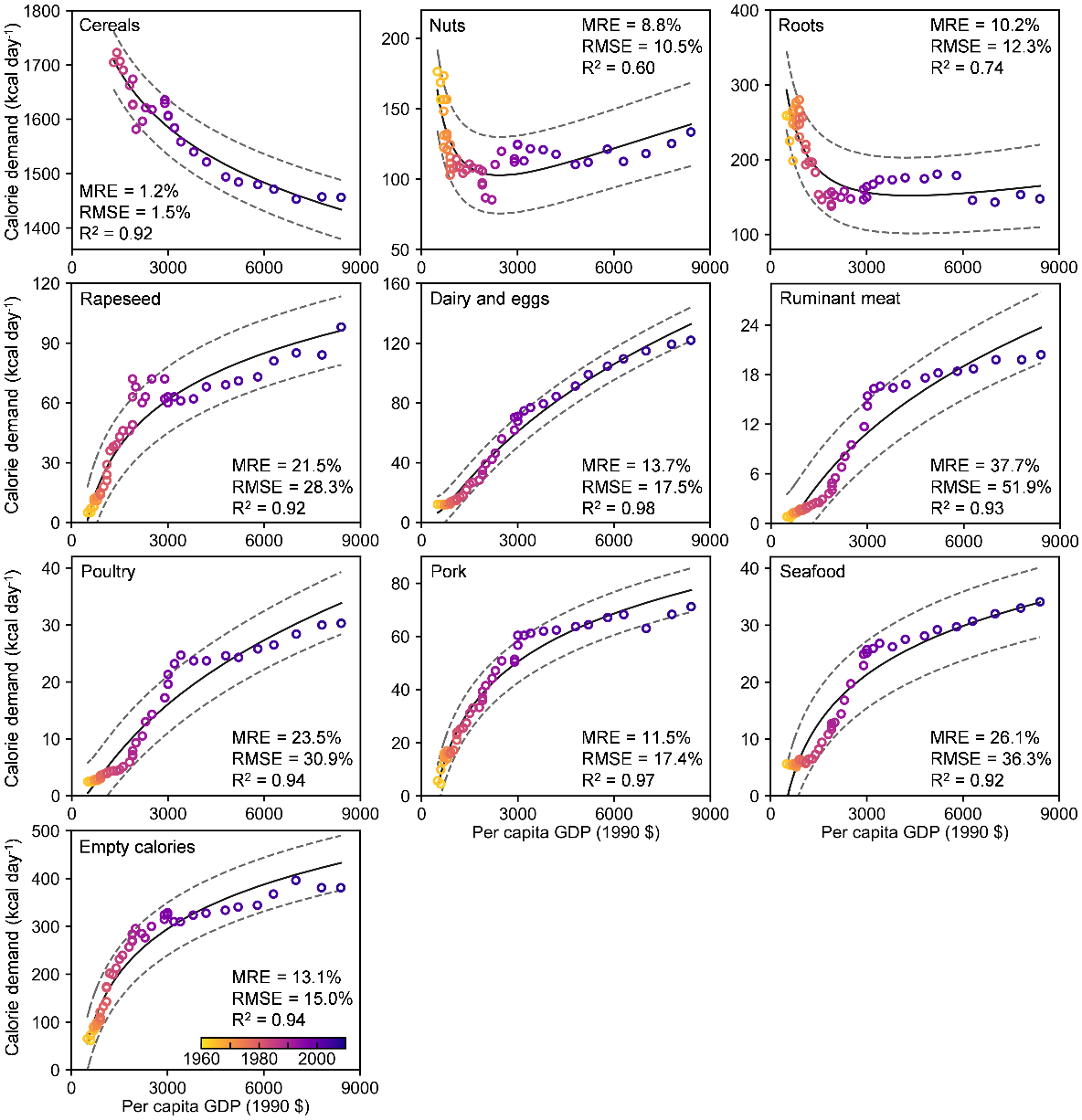
**

**Fig. S10 | Recalibration of empirical relationship between GDPs and calorie demand for each crop category in China using historical dataset from 1961 to 2009.**

**
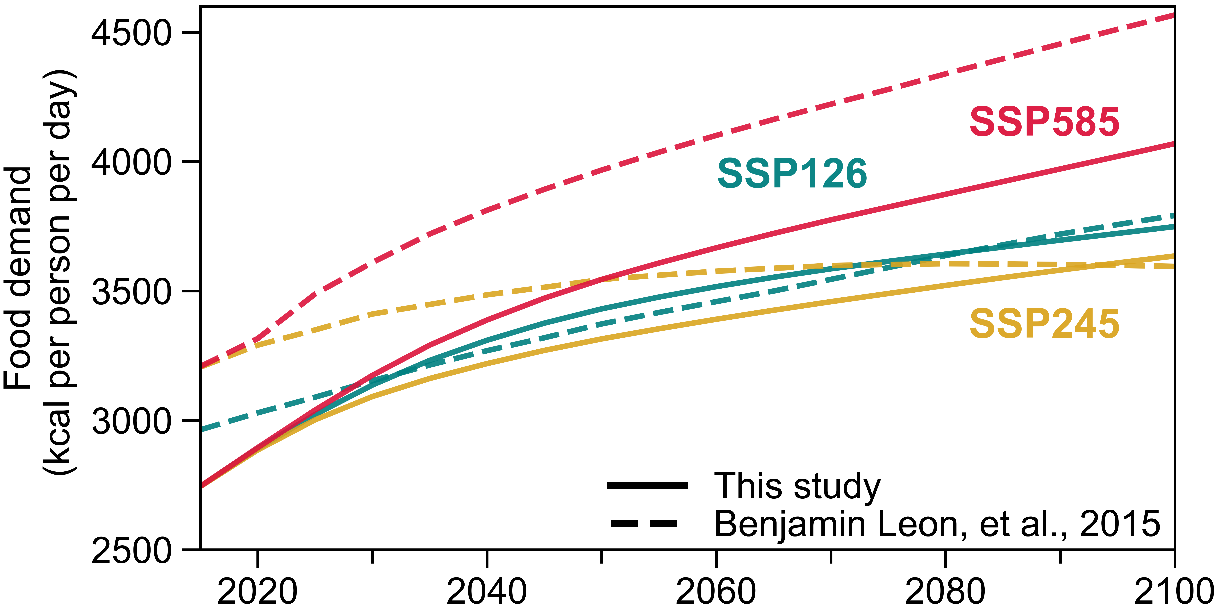
**

**Fig. S11 | Comparison of our predicted food caloric demand with previously estimates by numerical approaches (20).** Food caloric demand per person includes empty calories, four crop calories, and five animal calories, consistent with refs. For the details about predicting food demand for each category, please refer to Table S4 and Methods.


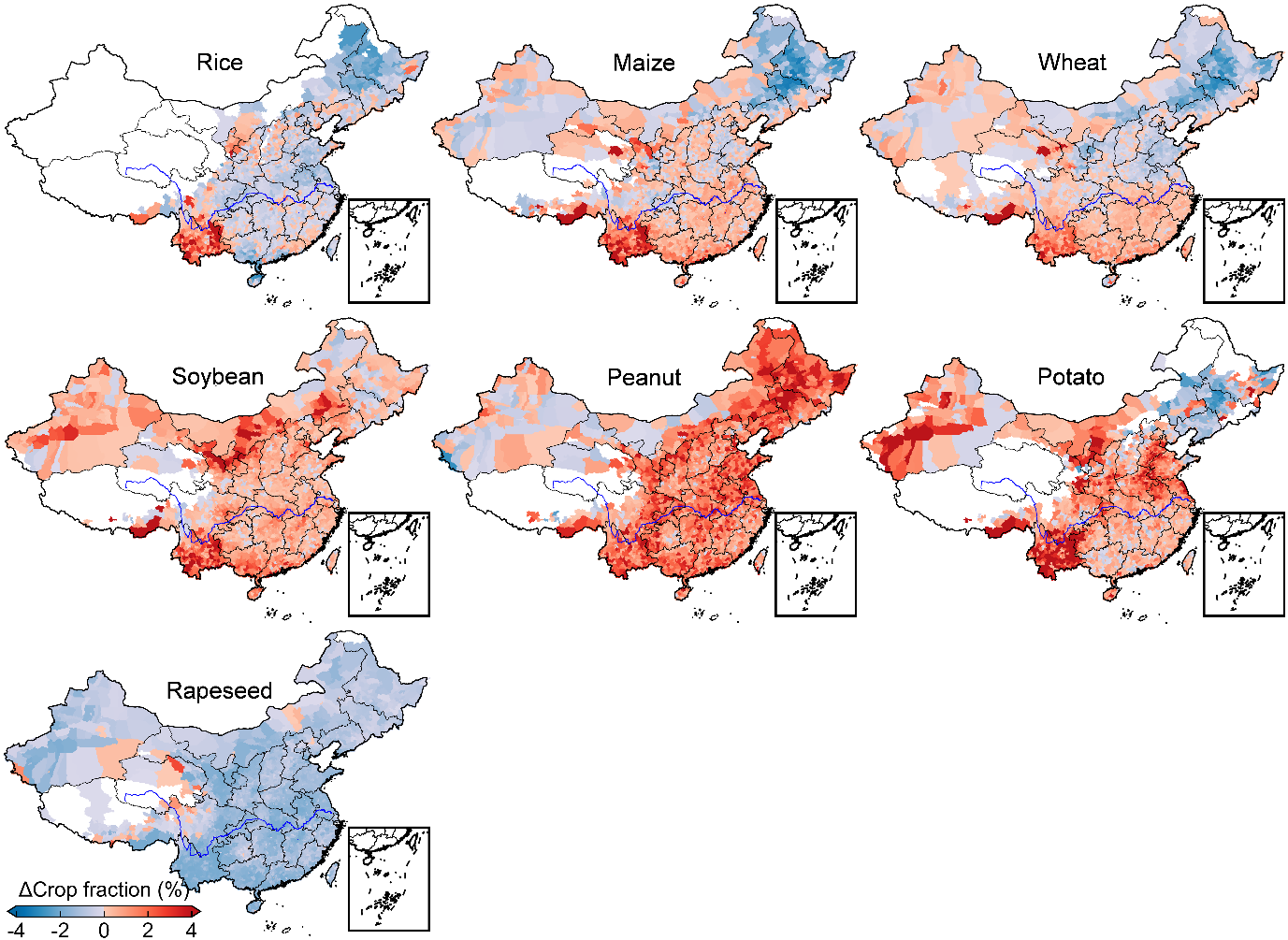


**Fig. S12 | Sensitivity analysis of the penalty factors in the multi-objective optimization.** Here we selected the SSP245 projected by MRI-ESM2-0 as the representative scenario to conduct the sensitivity analysis. Difference in county-based crop fractions were aggregated from 2020 to 2100.


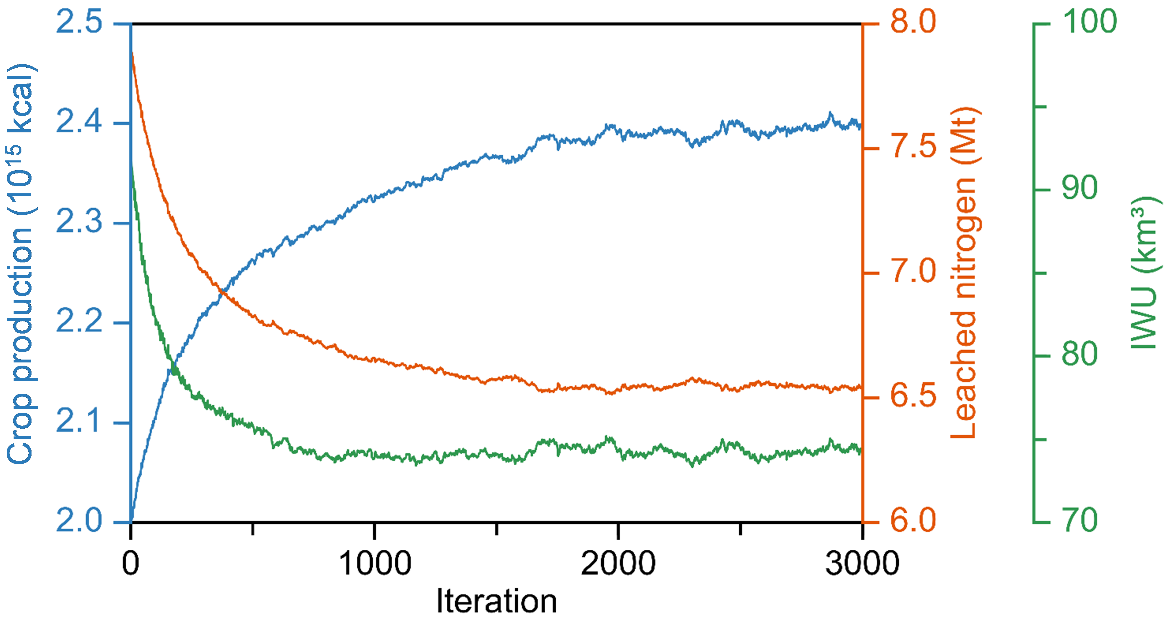


**Fig. S13 | Examples of iteration processes revealing the convergence of the NSGA-III optimization algorithm.** National crop production, leached nitrogen and irrigation water use are average values of all non-dominated solutions in each iteration in 2050 of SSP245 MRI-ESM2-0, where the maximum of iteration steps indicates sufficient to obtain the globally optimal solutions through 3000-generation evolution.

**References**

1. I. C. Prentice, Terrestrial nitrogen cycle simulation with a dynamic global vegetation model. *Global Change Biology* **14**, 1745-1764 (2008).

2. G. Y. Lu, D. W. Wong, An adaptive inverse-distance weighting spatial interpolation technique. *Computers & geosciences* **34**, 1044-1055 (2008).

3. M. Wang, C. Kroeze, M. Strokal, L. Ma, Reactive nitrogen losses from China's food system for the shared socioeconomic pathways (SSPs). *Science of the Total Environment* **605**, 884-893 (2017).

4. J. Wei, Z. Lee, S. Shang, A system to measure the data quality of spectral remote‐sensing reflectance of aquatic environments. *Journal of Geophysical Research: Oceans* **121**, 8189-8207 (2016).

5. W. Geng, L. Hu, J. Cui, M. Bu, B. Zhang, Biogas energy potential for livestock manure and gross control of animal feeding in region level of China. *Transactions of the Chinese Society of Agricultural Engineering* **29**, 171-179 (2013).

6. H. Müller Schmied *et al.*, The global water resources and use model WaterGAP v2. 2d: Model description and evaluation. *Geoscientific Model Development* **14**, 1037-1079 (2021).

7. K. Deb, H. Jain, An evolutionary many-objective optimization algorithm using reference-point-based nondominated sorting approach, part I: solving problems with box constraints. *IEEE transactions on evolutionary computation* **18**, 577-601 (2013).

8. J. Liu *et al.*, fSDE: efficient evolutionary optimisation for many-objective aero-engine calibration. *Complex & Intelligent Systems* **8**, 2731-2747 (2022).

9. G. Boccardo *et al.*, Development of a virtual methodology based on physical and data-driven models to optimize engine calibration. *Transportation Engineering* **10**, 100143 (2022).

10. W. Mkaouer *et al.*, Many-objective software remodularization using NSGA-III. *ACM Transactions on Software Engineering and Methodology (TOSEM)* **24**, 1-45 (2015).

11. M. W. Mkaouer, M. Kessentini, S. Bechikh, K. Deb, M. Ó Cinnéide (High dimensional search-based software engineering: finding tradeoffs among 15 objectives for automating software refactoring using NSGA-III. pp 1263-1270.

12. V. Yannibelli, E. Pacini, D. Monge, C. Mateos, G. Rodriguez, A comparative analysis of NSGA-II and NSGA-III for autoscaling parameter sweep experiments in the cloud. *Scientific Programming* **2020**, 1-17 (2020).

13. X. Sun, J. Fu, Many-objective optimization of BEV design parameters based on gradient boosting decision tree models and the NSGA-III algorithm considering the ambient temperature. *Energy* **288**, 129840 (2024).

14. S. M. Lim, A. B. M. Sultan, M. N. Sulaiman, A. Mustapha, K. Y. Leong, Crossover and mutation operators of genetic algorithms. *International journal of machine learning and computing* **7**, 9-12 (2017).

15. F. A. Zainuddin, M. F. Abd Samad, D. Tunggal, A review of crossover methods and problem representation of genetic algorithm in recent engineering applications. *International Journal of Advanced Science and Technology* **29**, 759-769 (2020).

16. M. Elarbi, S. Bechikh, A. Gupta, L. B. Said, Y.-S. Ong, A new decomposition-based NSGA-II for many-objective optimization. *IEEE transactions on systems, man, and cybernetics: systems* **48**, 1191-1210 (2017).

17. I. Das, J. E. Dennis, Normal-boundary intersection: A new method for generating the Pareto surface in nonlinear multicriteria optimization problems. *SIAM journal on optimization* **8**, 631-657 (1998).

18. Z. Y. Zhou, W. M. Tian, B. Malcolm, Supply and demand estimates for feed grains in China. *Agricultural Economics* **39**, 111-122 (2008).

19. D. Tilman, M. Clark, Global diets link environmental sustainability and human health. *Nature* **515**, 518-522 (2014).

20. B. L. Bodirsky *et al.*, Global food demand scenarios for the 21 st century. *PloS one* **10**, e0139201 (2015).
